# Colon Expression of Chemokines and Their Receptors Depending on the Stage of *Colitis* and Oat Beta-Glucan Dietary Intervention—Crohn’s Disease Model Study

**DOI:** 10.3390/ijms23031406

**Published:** 2022-01-26

**Authors:** Łukasz Kopiasz, Katarzyna Dziendzikowska, Joanna Gromadzka-Ostrowska

**Affiliations:** Department of Dietetics, Institute of Human Nutrition Sciences, Warsaw University of Life Sciences, Nowoursynowska 159c, 02-776 Warsaw, Poland; lukasz_kopiasz@sggw.edu.pl (Ł.K.); joanna_gromadzka_ostrowska@sggw.edu.pl (J.G.-O.)

**Keywords:** oat beta-glucan, *colitis*, Crohn’s disease, chemokines, chemokine receptors, osteopontin, rats

## Abstract

Crohn’s disease (CD), a condition characterized by chronic inflammation of the gastrointestinal tract with alternating periods of exacerbation and remission, is becoming common around the world. This study aimed to analyze the molecular mechanisms underlying the anti-inflammatory properties of oat beta-glucans of varying molar masses by modulating the expression of chemokines and their receptors as well as other proteins related to both stages of TNBS (2,4,6-trinitrobenzosulfonic acid)-induced *colitis*, which is an animal model of CD. The experiment involved 96 Sprague–Dawley rats, which were divided into two main groups: control and TNBS-induced *colitis*. Both groups of rats were further divided into three dietary subgroups, which were fed with standard feed or feed supplemented with low- or high-molar-mass oat beta-glucans for 3 (reflecting acute inflammation) or 7 days (reflecting pre-remission). The gene expression of chemokines and their receptors in the colon wall was determined by RT-PCR, and the expression of selected proteins in the mucosa was determined by immunohistochemical analysis. The results showed that acute and pre-remission stages of *colitis* were characterized by the increased gene expression of seven chemokines and four chemokine receptors in the colon wall as well as disrupted protein expression of CXCL1, CCL5, CXCR2, CCR5, and OPN in the mucosa. The consumption of oat beta-glucans resulted in decreased expression of most of these genes and modulated the expression of all proteins, with a stronger effect observed with the use of high-molar-mass beta-glucan. To summarize, dietary oat beta-glucans, particularly those of high molar mass, can reduce *colitis* by modulating the expression of chemokines and their receptors and certain proteins associated with CD.

## 1. Introduction

Crohn’s disease (CD) and ulcerative *colitis* (UC) belong to the group of nonspecific chronic inflammatory bowel diseases (IBD). IBD are multifactorial diseases that are characterized by interactions between genetic, microbiome, and environmental factors, which induce the development of inflammation by disrupting the immune response mechanisms [1]. In patients with CD, chronic inflammation can occur in any part of the gastrointestinal tract, covering the entire thickness of its wall, whereas in UC, it occurs in the mucosa and submucosa of the large intestine [2,3]. According to epidemiological data, the global incidence of IBD increased by 85% between 1990 and 2017, while the total number of IBD-related deaths during this period increased by 67%. Furthermore, IBD has been shown to affect millions of people around the world [4]. It should be noted that there is a significant link between IBD and other autoimmune diseases of the gastrointestinal system, including coeliac disease as well as IBD and colorectal cancer (CRC). Patients with coeliac disease have a >11-fold increased risk of developing IBD, while IBD patients have only a 2-fold increased risk of developing the coeliac disease [5]. On the other hand, patients with IBD have a high risk of CRC, which is a major cause of death in these patients. The risk of cancer development in IBD patients is time-dependent and can increase 18% by 30 years [6].

In CD, inflammation begins in the mucosa but can spread to the entire digestive tract (GT) wall over time. These pathological changes can affect the entire GT from the mouth to the rectum [7]. CD progresses through periods of exacerbation and remission. During an exacerbation, the immune cells are overstimulated, resulting in inflammatory infiltration of macrophages, T lymphocytes, neutrophils, and plasma cells [8]. The presence of inflammatory mediators, such as small chemotactic cytokines (chemokines), allows these immune cells to migrate to the developing site of inflammation [9].

The chemokines belong to the family of small proteins (8–10 kDa), which are divided into four subfamilies: C, CC, CXC, and CX3C. They act as potent chemoattractants for leukocyte activation and recruitment by interacting with G protein-linked transmembrane receptors on target cell membranes or atypical chemokine receptors, which regulate chemokine gradients and alleviate inflammation by scavenging chemokines in a G protein-independent manner [10,11]. In IBD, in addition to T lymphocytes, neutrophils, and macrophages, the intestinal wall is also infiltrated by other cells, namely fibroblasts, endothelial cells, and epithelial cells, which can themselves produce a variety of chemokines. Thus, chemokines and their receptors play an important role in inflammatory process through tissue-specific and immune cell-selective transport and retention of leukocytes at the site of inflammation. As a result, a significant inflammatory infiltrate forms during the periods of disease exacerbation [8]. Modulation of the production and secretion of tissue-specific chemokines can have an impact on the intensity and duration of inflammation.

Apart from chemotaxis, chemokines and their receptors are involved in various other functions, such as embryogenesis, angiogenic activity, and differentiation as well as functioning as helper T cells. Among others, CXCL1 and CXCL2 chemokines of the ELR subtype (containing Glu-Leu-Arg amino acid motif) exhibit angiogenic and chemotactic effects on neutrophils and are bound by the CXCR2 receptor. In contrast, chemokines from the same subfamily but of the non-ELR subtype, such as CXCL9, CXCL10, and CXCL11, modulate the activation and migration of T lymphocytes, NK cells, and monocytes by binding to the CXCR3 receptor [8,11,12,13]. Chemokines of CC type can attract different cell types such as neutrophils, eosinophils, macrophages, Treg, and Th1 and/or Th2 cells, as well as influence the differentiation of CD4+ cells [14]. In addition, each chemokine from this group performs specific functions: CCL3 and CCL4 act as macrophage inflammatory proteins, while CCL5 is actively involved in the recruitment of various immune cells (T lymphocytes, eosinophils, and basophils) [15,16]. CCL3, CCL4, and CCL5 exert their effects commonly through the CCR5 receptor, which is mainly expressed on macrophages as well as Th1 and NK cells. Moreover, these three chemokines act as ligands for the CCR1 receptor, which is expressed on macrophages, neutrophils, and basophils [17]. The chemokines analyzed in the present study are secreted from different cell types, namely intestinal epithelial cells (CCL3, CCL4, CCL5, CXCL1, CXCL2, CXCL9, CXCL10, CXCL11), macrophages (CXCL1, CCL5, CXCL10), mast cells (CCL5), eosinophils (CCL5), and neutrophils (CXCL1, CXCL2, CXCL10) [14,18,19]. The gene and/or protein expression of all these chemokines have been shown to be increased in patients suffering from CD and UC [14,20].

Other important factors involved in the pathogenesis of IBD are granulocyte colony-stimulating factor (G-CSF, also known as CSF3), oncostatin M (OSM), and osteopontin (OPN). CSF3 is essential for the granulocytopoiesis of neutrophils in the bone marrow and stimulates the proliferation of neutrophil precursors. CXCR2 and CXCR4 receptors mediate the retention of neutrophils in the bone marrow as well as their mobilization into the peripheral blood, for balancing the number of circulating neutrophils in the body [20]. Increasing the translocation of neutrophils to the site of inflammation leads to the release of these cells from the bone marrow, which increases the expression of CSF3 to stimulate granulocytopoiesis. OSM belongs to the IL-6 cytokine family and supports diverse homeostatic processes, including liver repair, osteoclastogenesis, and hematopoiesis [21]. Furthermore, this factor has been shown to bind extracellular matrix components such as collagen, fibronectin, and laminin. The overproduction of OSM is thought to promote various pathologies, including atherosclerosis, cancers, and inflammation of certain tissues, including inflammation associated with IBD [22]. OPN is a multifunctional glycoprotein that is also known as secreted phosphoprotein 1 (SPP1) and early T lymphocyte activation-1. OPN was found to regulate the development of T cells by increasing the differentiation of Th1 cells and suppressing the formation of Th2 cells. This protein has also been shown to be upregulated in UC and CD [23,24].

Beta-glucans are polysaccharides belonging to the dietary fiber fraction and composed of D-glucose monomers linked by β-1,3, β-1,4, or β-1,6 glycosidic bonds. They are commonly found in the cell wall of yeast, fungi, seaweed, and cereals such as oat and barley [25]. The cereal-derived beta-glucans have a linear structure and are linked by β-1,3 and β-1,4 glycosidic bonds, which allow them to dissolve well in water. Due to their specific structure, they are characterized by several functional and health-promoting properties, including having an anti-inflammatory effect [26,27].

This study aimed to understand the molecular mechanisms behind the anti-inflammatory action of oat beta-glucans of different molar masses by influencing the expression of chemokines and their receptors as well as other proteins related to both stages of *colitis* development. The study was conducted on a TNBS (2,4,6-trinitrobenzosulfonic acid)-induced model of inflammation, which is characteristic of human Crohn’s disease [28,29].

## 2. Results

### 2.1. Real-Time PCR Analysis of Gene Expression of Rat Inflammatory Cytokines, Chemokines, and Their Receptors

The results of the analysis performed using the Rat Inflammatory Cytokines and Receptors array showed that 3 days after TNBS administration, which corresponds to acute *colitis* stage, 23 out of 38 analyzed genes encoding chemokines and its receptors were upregulated in the *colitis* group (CβG−) compared to the healthy control group (HβG−). The upregulated genes included *Ccl2*, *Ccl3*, *Ccl4*, *Ccl7*, *Ccl12*, *Ccl17*, *Ccl19*, *Ccr1*, *Ccr2*, *Ccr3*, *Ccr4*, *Ccr5*, *Ccr8*, *Cxcl1*, *Cxcl2*, *Cxcl6*, *Cxcl9*, *Cxcr2*, *Cxcr3*, and *Cxcr5*. In addition, this list of genes included three other genes encoding important proteins related to inflammation response: *Osm*, *Spp1*, and *Csf3* (Table S1).

Consumption of feed with high-molar-mass oat beta-glucan (CβGh+ group) led to the upregulation of the expression of *Ccl20*, *Ccl24*, and *Cx3cl1* genes, and downregulation of the expression of *Ccl2*, *Ccl3*, *Ccl4*, *Ccl7*, *Ccl19*, *Ccr1*, *Ccr2*, *Ccr5*, *Cxcl1*, *Cxcl2*, *Cxcl6*, *Cxcr1*, *Cxcr2*, *Csf3*, *Osm*, and *Spp1*. Significant dietary effects were also observed in animals with induced *colitis* that were fed a diet supplemented with low-molar-mass oat beta-glucan (CβGl+). Rats from this group showed an upregulated expression of genes encoding *Cxcl9*, *Cxcl10*, and *Cxcl11* and downregulated expression of genes encoding *Ccl3*, *Ccl4*, *Ccl19*, *Ccl22*, *Ccr1*, *Ccr4*, *Ccr8*, *Cxcl2*, *Cxcr1*, *Cxcr2*, *Cxcr3*, *Cxcr5*, *Csf3*, and *Osm* (Table S1).

After 7 days of induced *colitis*, upregulation of the expression of 22 genes (*Ccl2*, *Ccl3*, *Ccl4*, *Ccl5*, *Ccl6*, *Ccl7*, *Ccl12*, *Ccl17*, *Cxcl1*, *Cxcl2*, *Cxcl6*, *Cxcl9*, *Cxcl11*, *Ccr1*, *Ccr2*, *Ccr3*, *Ccr5*, *Ccr8*, *Cxcr2*, *Csf3*, *Osm*, and *Spp1*) was observed in the CβG− group compared to the HβG- control group. On the other hand, *colitis* rats receiving feed with high-molar-mass oat beta-glucan (CβGh+) resulted in higher expression of only four genes (*Ccl19*, *Ccr4*, *Ccr8*, *Cxcl9*). Similarly, *colitis* rats receiving feed with low-molar-mass oat beta-glucan (βGl+) upregulated the expression of only four genes (*Ccl19*, *Cxcl10*, *Cx3cr1*, *Cxcr5*) in this stage of *colitis* (Table S1).

Based on these results and literature review, we selected the following chemokines and their receptors (*Ccl3*, *Ccl4*, *Ccl5*, *Cxcl1*, *Cxcl2*, *Cxcl9*, *Cxcl10*, *Cxcl11*, *Ccr1*, *Ccr2*, *Ccr5*, *Cxcr2*), which exhibit a significant role in the development of colon inflammation for further investigation. The results of the expression of all assessed chemokine genes and their receptors are presented in the Supplementary Materials in Tables S2 and S3.

**Figure 1:**
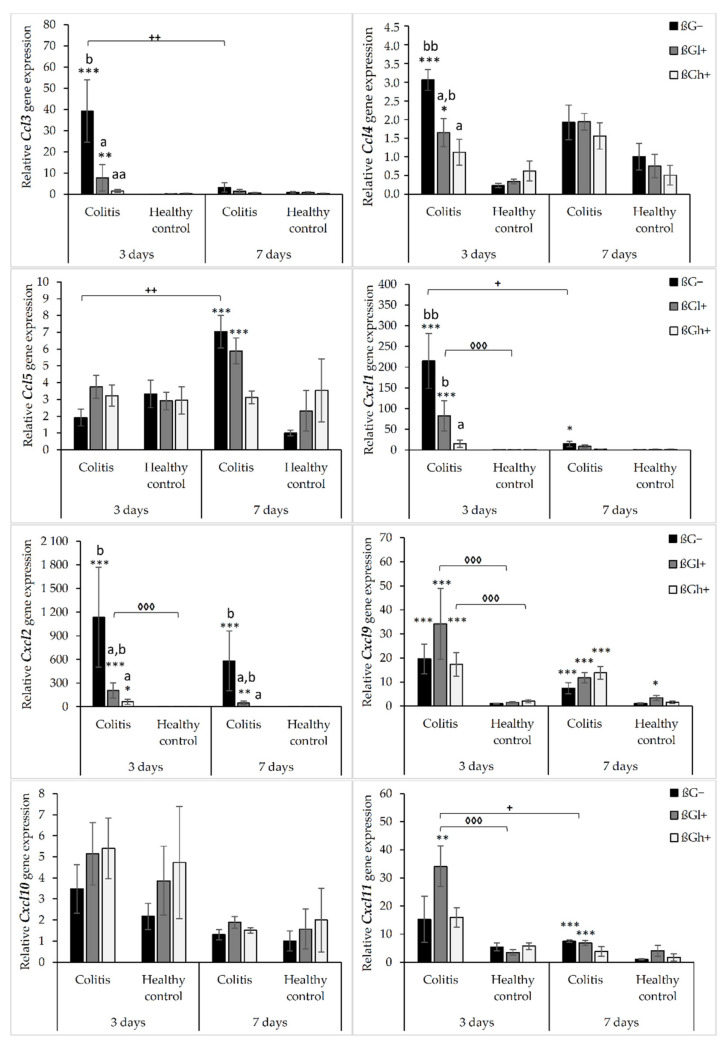
Relative gene expression of chemokines. * Significantly different from the control group (healthy control βG−) at the same time point according to the Dunnett post hoc test (* *p* < 0.05, ** *p* < 0.01, *** *p* < 0.001). ^◊^ Significantly different between the *colitis* and control groups at the same time point and with the same feed according to the Tukey post hoc test (^◊◊◊^ *p* < 0.001). ^+^ Significantly different from the same subgroup at another time point according to the Tukey post hoc test (^+^ *p* < 0.05, ^++^ *p* < 0.01). ^a,b^ Different letters denote significant differences in the *colitis*/control group at the same time point according to the Tukey post hoc test (^a,b^ *p* < 0.05, ^aa,bb^ *p* < 0.01).

The results from the analysis of expression of chemokine genes are presented in Figure 1. ANOVA showed that the gene expression of all the investigated chemokines (except for Cxcl10) was significantly higher in the *colitis* group (C) than in the control group (H) (*p* < 0.01, Figure S1). Furthermore, the gene expression of *Ccl3*, *Ccl4*, *Cxcl1*, and *Cxcl2* was significantly lower in the group that received feed with high-molar-mass oat beta-glucan (βGh+) compared to the rats consuming control diet without beta-glucans (βG−) (ANOVA and Tukey post hoc, *p* < 0.05, Figure S2). Compared to 3 days of TNBS administration, a significant reduction in the gene expression of *Ccl3*, *Cxcl1*, *Cxcl2*, *Cxcl10*, and *Cxcl11* was observed after 7 days (ANOVA, *p* < 0.05, Figure S3). Moreover, as was shown by ANOVA, a significant interaction was noticed between colon inflammation and dietary intervention, which influenced the gene expression of *Ccl3*, *Cxcl1*, and *Cxcl2* (*p* < 0.01, Figure S4). Significantly higher gene expression of these chemokines was observed in the CβG− group compared to the control group (Tukey post hoc, *p* < 0.001), while in the CβGh+ group, the gene expression was lower compared to the CβG− group (*p* < 0.001) and at the same level as in the control group. Additionally, ANOVA revealed a statistically significant interaction between all three experimental factors (colon inflammation, dietary intervention, and period of its supplementation), which influenced the gene expression of *Ccl3*, *Ccl4*, *Ccl5*, and *Cxcl11* (*p* < 0.05). Moreover, the gene expression of all selected chemokines and their receptors in the groups without induced *colitis* was low and consistent.

The gene expression of *Ccl3* and *Ccl4* chemokines was significantly higher in the CβG− group compared to the control group (HβG−) only 3 days after TNBS administration. In CβGl+ and CβGh+ groups, the gene expression of these chemokines was lower compared to the CβG− group, but in rats from the CβGh+ group, a greater reduction in the gene expression of *Ccl3* and *Ccl4* was observed (Figure 1).

Only after 7 days of TNBS administration, the gene expression of *Ccl5* was increased in the *colitis* group receiving feed without beta-glucan (βG−) compared to the control group (HβG−). In addition, at this time point, *Ccl5* gene expression in the CβGh+ group was at a similar level as in the control group (HβG−).

The gene expression of *Cxcl1* and *Cxcl2* was higher in the CβG− group than in the control group (HβG−) after 3 and 7 days of TNBS administration. Moreover, the gene expression of these chemokines in the CβGh+ group was at a similar level as in the control group (HβG−) at both time points. The gene expression of Cxcl9 was higher in all *colitis* groups compared to the control group (HβG−) after both 3 and 7 days of TNBS administration without any dietary intervention effect. Furthermore, the gene expression of *Cxcl11* in the *colitis* group consuming feed with beta-glucan (βGl+) was higher compared to the control group (HβG−) at both time points. However, after 7 days of *colitis* induction, the gene expression of *Cxcl11* was lower in the CβGl+ group.

**Figure 2:**
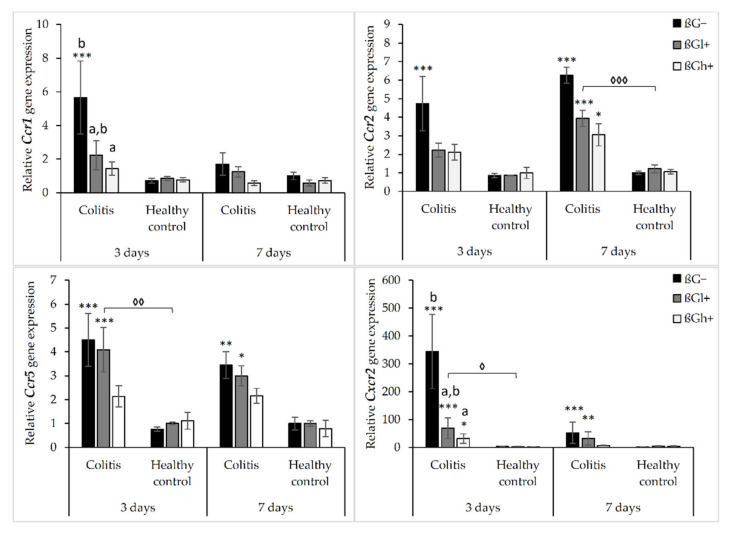
Relative gene expression of chemokine receptors. * Significantly different from the control group (healthy control βG−) at the same time point according to the Dunnett post hoc test (* *p* < 0.05, ** *p* < 0.01, *** *p* < 0.001). ^◊^ Significantly different between the *colitis* and control groups at the same time point and with the same feed according to the Tukey post hoc test (^◊^ *p* < 0.05, ^◊◊^ *p* < 0.01, ^◊◊◊^ *p* < 0.001). ^a,b^ Different letters denote significant differences in the *colitis*/control group at the same time point according to the Tukey post hoc test (^a,b^ *p* < 0.05).

The results from the analysis of expression of chemokine receptor genes are presented in Figure 2. As shown by ANOVA, the gene expression of *Ccr1*, *Ccr2*, *Ccr5*, and *Cxcr2* was higher in the rats with TNBS-induced *colitis* than in the control group (*p* < 0.001, Figure S5). ANOVA showed that the gene expression of *Ccr1* and *Ccr2* was significantly lower in the group consuming feed with high-molar-mass oat beta-glucan (βGh+) compared to the rats consuming a diet without beta-glucan (βG−) (*p* < 0.05, Figure S6). Moreover, compared to 3 days of dietary intervention, *Ccr1* gene expression was significantly lower after 7 days, while *Ccr2* gene expression was higher (ANOVA, *p* < 0.05, Figure S7). A statistically significant interaction between colon inflammation and dietary intervention was observed, which influenced the gene expression of *Ccr2* and *Cxcr2* (ANOVA, *p* < 0.01). The same trend was found in the case of the gene expression of *Ccr1* and *Ccr5* (ANOVA, *p* = 0.07 for *Ccr1*; *p* = 0.085 for *Ccr5*). A significantly higher expression of both these genes was observed in the CβG− group compared to the control group (HβG−) (*p* < 0.01), while in the CβGh+ group, the gene expression of all these receptors was decreased compared to the CβG− group (Tukey post hoc, *p* < 0.05) (Figure S8).

The gene expression of *Ccr2*, *Ccr5*, and *Cxcr2* was increased in *colitis* rats fed with feed without beta-glucan (βG−) compared to the control group (HβG−) at both time points, whereas *Ccr1* gene expression was increased only after 3 days of TNBS administration. The gene expression of these chemokine receptors (*Ccr2*, *Ccr5*, and *Cxcr2*) in the *colitis* group fed with feed containing high-molar-mass oat beta-glucan (CβGh+) was significantly lower compared to the CβG− group and was at a similar level as in the control group (HβG−) at both time points. The same results were observed for *Ccr1* after 3 days of dietary intervention. Furthermore, after 3 days of *colitis* induction, the gene expression of *Ccr1* and *Ccr2* was significantly decreased in the animals from the CβGl+ group compared to the rats from the CβG− group. Summing up, the consumption of feed with 1% of high-molar-mass oat beta-glucan decreased the gene expression of *Ccr2*, *Ccr5*, and *Cxcr2* in *colitis* rats at both time points, while the consumption of feed with low-molar-mass oat beta-glucan decreased the gene expression of *Ccr1* and *Ccr2* only after 3 days of *colitis* induction.

In the control groups, the gene expression of *Ccr1*, *Ccr2*, *Ccr5*, and *Cxcr2* did not change significantly depending on both inflammation and dietary intervention using oat beta-glucans regardless of their molar mass (Figure 2).

**Figure 3:**
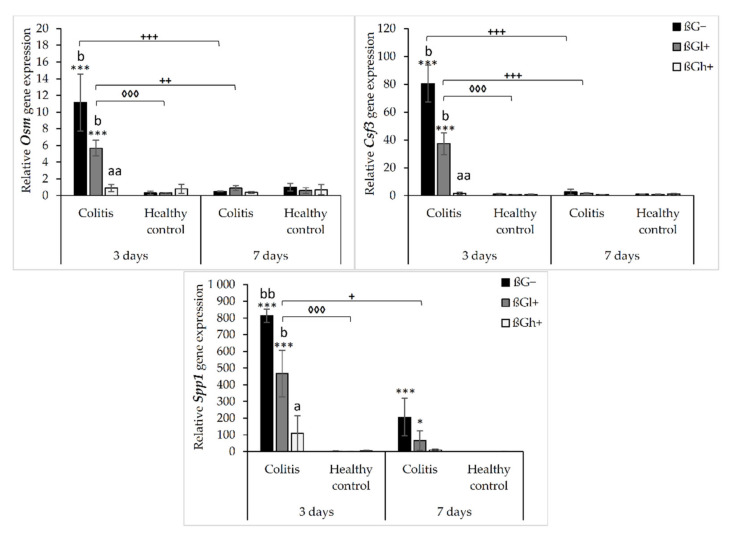
Relative gene expression of *Osm*, *Csf3*, and *Spp1*. * Significantly different from the control group (healthy control βG−) at the same time point according to the Dunnett post hoc test (* *p* < 0.05, *** *p* < 0.001). ^◊^ Significantly different between the *colitis* and control groups at the same time point and with the same feed according to the Tukey post hoc test (^◊◊◊^ *p* < 0.001). ^+^ Significantly different from the same subgroups at another time point according to the Tukey post hoc test (^+^ *p* < 0.05, ^++^ *p* < 0.01, ^+++^ *p* < 0.001). ^a,b^ Different letters denote significant differences in the *colitis*/control group at the same time point according to the Tukey post hoc test (^a,b^ *p* < 0.05, ^aa,bb^ *p* < 0.01).

The relative gene expression of other colon inflammation-related proteins (*Osm*, *Csf3*, and *Spp1*) which increased after *colitis* induction are presented in Figure 3. ANOVA showed a significant influence of all three experimental factors (colon inflammation, dietary intervention, and period of its use) on the gene expression of *Osm*, *Csf3*, and *Spp1*. Analysis of each factor separately showed that the gene expression of these proteins increased in rats with induced *colitis* (*p* < 0.001, Figure S9), while the consumption of feed with high-molar-mass oat beta-glucan resulted in a significantly lower gene expression of *Osm* and *Csf3* compared to the rats consuming βG− feed (ANOVA, *p* < 0.01, Tukey post hoc, *p* < 0.05, Figure S10). Moreover, the gene expression of all these three proteins at the 7 days time point was decreased compared to the 3 days time point (*p* < 0.001, Figure S11). Importantly, ANOVA showed an interaction between induced colon inflammation and dietary intervention with feed containing oat beta-glucans, which statistically significantly influenced the gene expression of *Osm*, *Csf3*, and *Spp1* (*p* < 0.001, Figure S12). The gene expression of all these three proteins in the *colitis* group fed with feed without beta-glucans (CβG−) was significantly higher compared to the control group (HβG−) (Tukey post hoc, *p* < 0.01), while in the *colitis* group fed with feed containing high-molar-mass oat beta-glucan (CβGh+), the gene expression of these proteins was at a similar level as in the control group. In addition, ANOVA showed an interaction between all three experimental factors, which influenced the gene expression of *Osm* and *Csf3* (*p* < 0.001).

After 3 days of induced inflammation, the gene expression of *Osm*, *Csf3*, and *Spp1* was higher in the CβG− group compared to the control group (HβG−), while the gene expression of all these three proteins in the rats from the CβGh+ group was at a similar level as in the HβG− group and significantly lower compared to the CβG− group. After 7 days of induced *colitis*, the gene expression of *Osm* and *Csf3* was at the same level in all experimental groups, while the gene expression of *Spp1* was still higher in CβG− and CβGl+ groups compared to the HβG− group. In all control groups, no changes were observed in the gene expression of these proteins (Figure 3).

### 2.2. Expression of Selected Chemokines and Their Receptors in the Colon Mucosa

**Figure 4:**
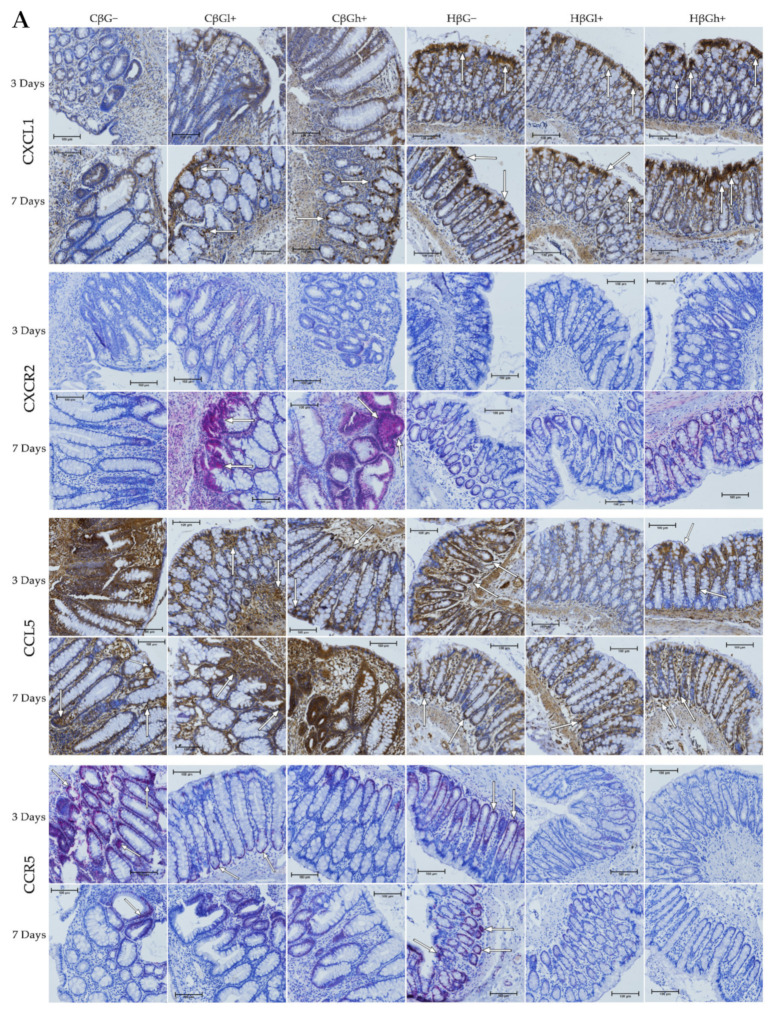

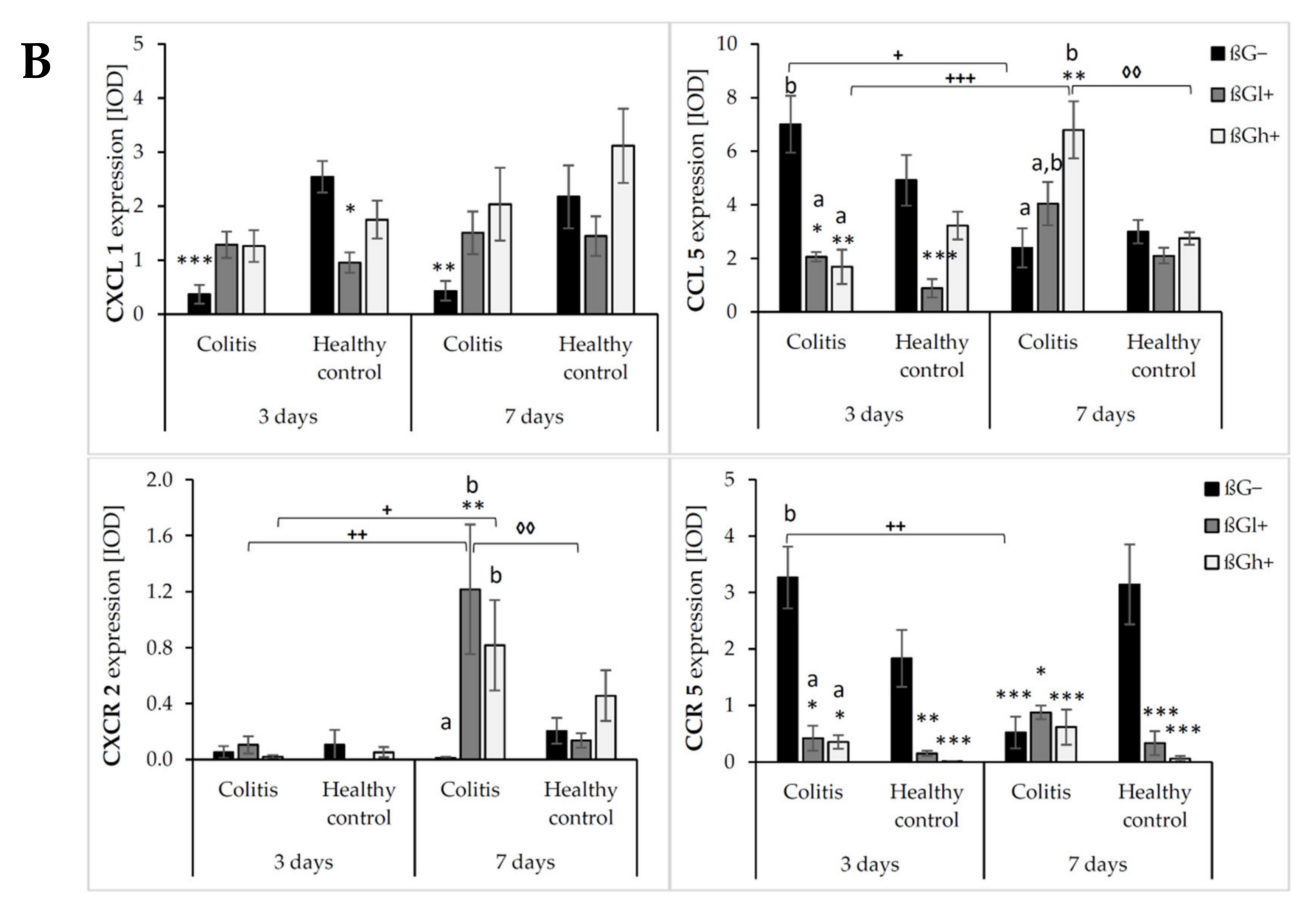
Light micrographs imaged under the NIKON Eclipse Ti2 microscope (**A**)**.** White arrows indicate mucosa and/or immune cells with high expression of CXCL1, CXCL5, CXCR2 and CCR5 proteins. Expression of chemokines CXCL1 and CCL5 and their receptors CXCR2 and CCR5 in the colon mucosa: results of the immunohistochemical analysis (**B**). * Significantly different from the control group (control βG−) at the same time point according to the Dunnett post hoc test (* *p* < 0.05, ** *p* < 0.01, *** *p* < 0.001). ^◊^ Significantly different between the *colitis* and control groups at the same time point and with the same feed according to the Tukey post hoc test (^◊◊^ *p* < 0.01). ^+^ Significantly different from the same subgroups at another time point according to the Tukey post hoc test (^+^ *p* < 0.05, ^++^ *p* < 0.01, ^+++^ *p* < 0.001). ^a,b^ Different letters denote significant differences in the *colitis*/control group at the same time point according to the Tukey post hoc test (^a,b^ *p* < 0.05).

The results from the immunohistochemical analysis of the expression of selected chemokines (CXCL1 and CCL5) and their receptors (CXCR2 and CCR5) in the colon mucosa are presented in Figure 4. ANOVA showed that the occurrence of inflammation had a significant influence on the expression of both chemokines and CXCR2 (*p* < 0.01 for CCL5; *p* < 0.001 for CXCL1; *p* < 0.05 for CXCR2, Figure S13). In the *colitis* group, the expression of CXCL1 was lower and the expression of CCL5 and CXCR2 was higher compared to the control group. Moreover, the consumption of oat beta-glucans influenced the expression of these chemokines and their receptors (ANOVA, *p* < 0.01, Figure S14). Compared to the other dietary groups, the expression of CXCL1 in the rats fed with feed containing high-molar-mass oat beta-glucan (βGh+) was higher (Tukey post hoc, *p* < 0.05), while the expression of CCL5 was lower in the βGl+ group (Tukey post hoc, *p* < 0.05 for βGh+ and *p* < 0.01 for βG−, Figure S14A). In the rats from the βGh+ and βGl+ groups, CXCR2 expression was higher compared to the rats from the βG− group (Tukey post hoc, *p* < 0.05), while CCR5 expression in the βGl+ and βGh+ groups was lower compared to the βG− group (Tukey post hoc, *p* < 0.001, Figure S14B). Time point after *colitis* induction only had an influence on CXCR2 expression; the expression of this protein after 7 days of dietary intervention was higher compared to 3 days (ANOVA, *p* < 0.001, Figure S15).

Moreover, ANOVA showed a significant interaction between colon inflammation and dietary intervention, which influenced the protein expression of CXCL1 (*p* < 0.001, Figure S16A), CXCR2, and CCR5 (*p* < 0.01, Figure S16B). CXCL1 expression in the rats from the *colitis* group fed with feed without beta-glucans (CβG−) was higher compared to the rats from the control group receiving the same feed (HβG−) (Tukey post hoc, *p* < 0.001), whereas in the *colitis* rats receiving feed with low- (βGl+) or high-molar-mass oat beta-glucan (βGh+), significantly higher expression of CXCL1 than the CβG− group and a similar level of expression to the control group (HβG−) were observed (Tukey post hoc, *p* < 0.01). Furthermore, CXCL1 expression in the HβGl+ group was lower compared to the HβGh+ group (Tukey post hoc, *p* < 0.05, Figure S16A). CXCR2 expression increased in the CβGl+ group compared to the CβG− group (Tukey post hoc, *p* < 0.05), but the expression did not significantly differ compared to the control groups (Figure S16B). On the other hand, CCR5 expression was lower in the βGl+ and βGh+ dietary subgroups with and without induced *colitis* (CβGl+, CβGh+, HβGl+, and HβGh+) compared to the control βG− group (Tukey post hoc, *p* < 0.001, Figure S16B).

After 3 and 7 days of TNBS administration, the expression of CXCL1 decreased in the rats with induced *colitis* fed with feed without beta-glucans (CβG−) compared to the control group (HβG−), while in the *C*βGl+ and CβGh+ groups, the expression level of this chemokine was at a similar level as in the control group (HβG−).

ANOVA showed an interaction between all three experimental factors, which influenced the expression level of CCL5 and CCR5 (*p* < 0.001). After 3 days of induced *colitis*, the expression of CCL5 was significantly higher in the CβG− group compared to CβGl+ and CβGh+ groups, whereas statistically significant lower expression of this chemokine was observed in CβGl+ and CβGh+ groups compared to the control group (HβG−). Additionally, the expression of CCL5 was reduced in the HβGl+ group compared to the control group (HβG−). However, after 7 days of TNBS administration, the expression of CCL5 protein differed between the experimental groups. In the *colitis* group fed with feed containing high-molar-mass oat beta-glucan (βGh+), a higher expression of CCL5 was observed compared to the control group (HβG−) and the *colitis* group fed with feed without beta-glucan (βG−). Moreover, the expression of this chemokine was statistically significantly lower in the CβG− group and higher in the CβGh+ group after 7 days of TNBS administration compared to that observed after 3 days.

After 3 days of induced *colitis*, a lower level of CCR5 expression was observed in the CβGl+, CβGh+, HβGl+, and HβGh+ groups compared to the control group (HβG−), with the lowest level of expression in the rats from the HβGh+ group. Moreover, in the CβG− group, a higher level of expression of this chemokine receptor was observed compared to CβGl+ and CβGh+ groups. After 7 days of TNBS administration, the expression of this chemokine receptor in the HβG− group was still at the same level as in the HβG− group observed after 3 days, while in the CβG− group, the level decreased compared to the level observed in CβG− after 3 days. In addition, after 7 days of induced *colitis*, the expression of CCR5 was more than 3-fold higher in the HβG− group compared to the other experimental groups at this time point.

ANOVA showed some trend of interaction between all three experimental factors (*p* = 0.051) for CXCR2 expression, whereas post hoc analysis indicated statistically significant differences between the experimental groups only after 7 days of TNBS administration. At this time point, increased expression of CXCR2 protein was found in the rats from the CβGl+ and CβGh+ groups compared to the CβG− group. Moreover, the consumption of feed containing low-molar-mass oat beta-glucan resulted in the highest increase in CXCR2 expression in the rats with induced *colitis* compared to the feed containing high-molar-mass oat beta-glucan.

**Figure 5:**
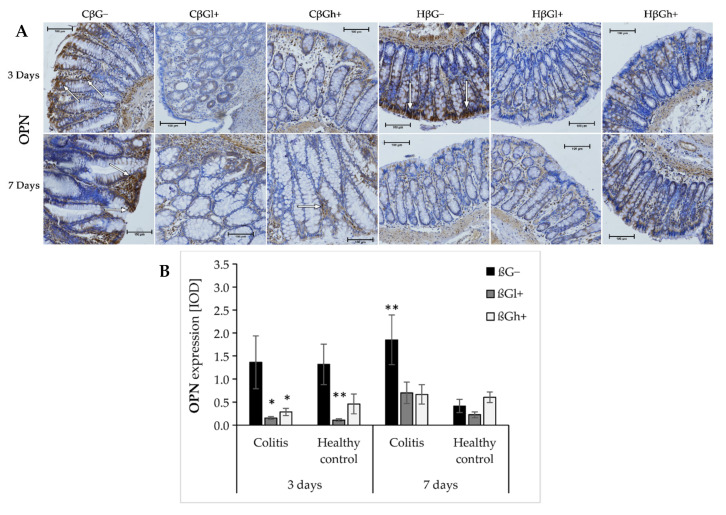
Light micrographs imaged under the NIKON Eclipse Ti2 microscope (**A**). White arrows indicate mucosa and/or immune cells with high expression of OPN proteins; Expression of OPN in the colon mucosa: results of the immunohistochemical analysis (**B**). * Significantly different from the control group (control βG−) at the same time point according to the Dunnett post hoc test (* *p* < 0.05, ** *p* < 0.01).

The results from the analysis of expression of OPN are presented in Figure 5a,b. ANOVA showed that the consumption of feed supplemented with oat beta-glucans had a significant influence on OPN expression (*p* < 0.001). In the groups fed with βGl+ and βGh+ feed, the expression of OPN was lower compared to the group fed with feed without beta-glucans (βG−) (Tukey post hoc, *p* < 0.01, Figure S17). After 3 days of *colitis* induction, OPN expression was decreased in the *colitis* groups fed with feed containing low- or high-molar-mass beta-glucan (CβGl+/CβGh+) and the healthy control group fed with feed containing low-molar-mass beta-glucan (HβGl+) compared to the control group (HβG−). However, after 7 days of *colitis* induction, higher OPN expression was observed only in the CβG− group compared to the HβG− group, while in CβGl+ and CβGh+ groups, the expression of this protein was at a similar level as in the control group.

### 2.3. Fisher’s Linear Discriminant Analysis (FLD)

**Figure 6:**
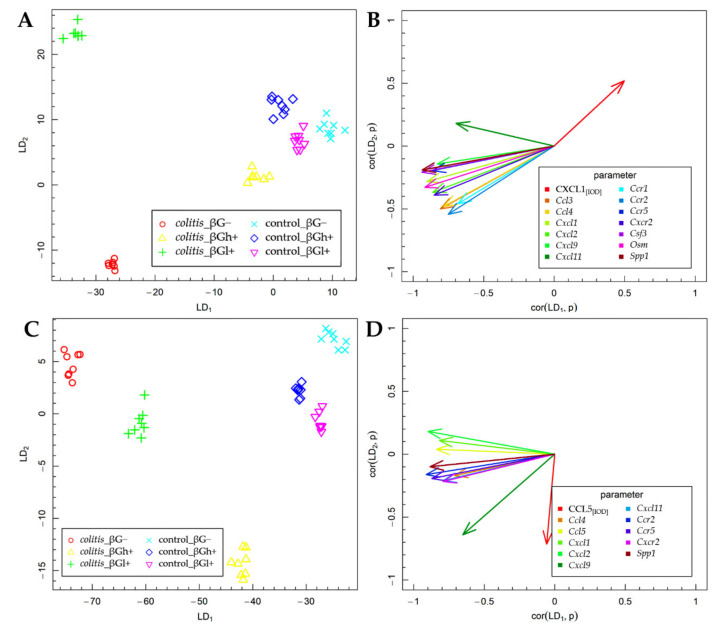
Fisher’s Linear Discriminant (FLD) analysis: (**A**,**C**) experimental data on the plane spanned by two of the most data-separating FLDs and (**B**,**D**) parameters contributing the most to FLDs. (**A**,**B**) 3 days; (**C**,**D**) 7 days.

The results of the FLD analysis for the expression of experimental factors are presented in Figure 6. The FLD analysis was used to identify the linear combinations of gene and protein of the analyzed chemokines and their receptors and three other colon inflammation-related proteins that allow the best separation of the rat groups at the two time points considered in the experimental model. The experimental data were divided into two groups based on the time points. The method provides a linear combination of parameters used in the analysis, such that the separation of data groups is the best possible among all linear combinations of parameters. The data groups at the two time points considered in the study are shown in Figure 6A,C. Six experimental groups are distinguished in each figure. The data are presented in the space spanned by linear combinations of parameters (LDs), marked as LD_1_ and LD_2_, which are the best separating those predefined groups.

The vectors visible in Figure 6B,D indicate the direction in which relevant parameters determine the separation of experimental groups at two time points—3 days (Figure 6A,B) and 7 days (Figure 6C,D). High values of the parameter corresponding to a particular vector caused the shift of the data to the direction determined by the vector. The FLD analysis complemented ANOVA and allowed us to sum up the obtained results.

The FLD analysis of the animal parameters that was performed for data collected after 3 days of TNBS or saline solution administration (Figure 6A,B) showed that the factors that most differentiated these experimental groups were the gene expression of *Ccl3*, *Ccl4*, *Cxcl1*, *Cxcl2*, *Cxcl9*, *Cxcl11*, *Ccr1*, *Ccr2*, *Ccr5*, *Cxcr2*, *Csf3*, *Osm*, and *Spp1* and the protein expression of CXCL1. The results of this analysis showed that it was possible to determine a combination of the above parameters, which allows separating *colitis* βG− and βGl+ groups from *colitis* βGh+ and control groups in a horizontal plane (LD_1_). The vertical plane (LD_2_) enabled the separation of the CβG− group from the CβGl+ group as well as from the other groups (CβGh+, HβG−, HβGl+, and HβGh+) (Figure 6A). The gene expression of all previously mentioned parameters was the most correlated with LD_1_, while the protein expression of CXCL1 in the colon mucosa was important in the case of both LDs (Figure 6B). Thus, it can be concluded that the CβG− group was characterized by a significantly higher gene expression of almost all the above chemokines and their receptors (except for *Cxcl11*) compared to the CβGh+ and control groups, while in the CβGl+ group, the expression of the analyzed chemokines and their receptors was at an intermediate level. Moreover, the CβGl+ group showed the highest gene expression of *Cxcl11*. The direction of the CXCL1 vector indicates the highest expression in control groups and CβGh+ group and the lowest expression in the CβG− group. This clearly shows an inverse correlation between the gene expression of *Cxcl1* and the protein expression of this chemokine.

After 7 days of *colitis* induction (Figure 6C,D), the horizontal plane (LD_1_) enabled the separation of *colitis* groups from control groups, as well as the separation of *colitis* dietary subgroups from each other (CβG−, CβGl+, and CβGh+ group). Animals from the *colitis* group fed with feed containing high-molar-mass oat beta-glucan (βGh+) were separated from the other groups in the vertical plane (LD_2_) (Figure 6C). The gene expression of *Ccl4*, *Ccl5*, *Cxcl1*, *Cxcl2*, *Cxcl11*, *Ccr2*, *Ccr5*, *Cxcr2*, and *Spp1* had the greatest influence on LD_1_, while CCL5 expression had the most impact on LD_2_. Both LDs were influenced by the gene expression of *Cxcl9* (Figure 6D). Thus, it can be concluded that the higher gene expression of *Ccl4*, *Ccl5*, *Cxcl1*, *Cxcl2*, *Cxcl11*, *Ccr2*, *Ccr5*, *Cxcr2*, and *Spp1* was still observed in the CβG− group compared to the control groups. Moreover, rats from *colitis* groups fed with feed containing low- (βGl+) or high-molar-mass oat beta-glucan (βGh+) showed the lower expression of previously mentioned parameters compared to the CβG− group, with the greatest effect observed with the feed supplemented with high-molar-mass oat beta-glucan. The CCL5 expression was increased only in the CβGh+ group compared to other groups. Furthermore, this analysis showed that induced *colitis* caused an increase in *Ccl5* gene expression but did not influence the expression of CCL5, while the consumption of feed with high-molar-mass oat beta-glucan increased CCL5 protein expression and decreased *Ccl5* gene expression in rats with *colitis* compared to the rats from the CβG group.

## 3. Discussion

Crohn’s disease, a type of IBD, is characterized by periods of exacerbation and remission. Exacerbation periods are accompanied by significant leukocyte inflammatory infiltration of the intestinal mucosa and submucosa, including macrophages, neutrophils, and T lymphocytes (such as Th1 and Th17 cells). These immune cells migrate by binding integrin molecules located on the surface of leukocytes to cellular adhesion molecules expressed on the surface of endothelial cells. Moreover, in response to inflammatory signals in the intestinal mucosa, endothelial cells from intestinal blood vessels and intestinal epithelial cells produce chemokines, which act as chemoattractants for leukocytes [8,30]. The TNBS model of *colitis* used in this study is characteristic of CD in humans, due to the presence of transmural inflammatory lesions with dense infiltration of lymphocytes (mainly Th1) and the secretion of proinflammatory cytokines in the entire intestinal wall [28,29]. Acute inflammation observed after 3 days of TNBS administration is caused by colon cell damage. Molecules from damaged cells act as chemotactic elements, stimulating the migration of immune cells from the blood to the inflamed tissue. The recruited immune cells increase the expression of proinflammatory cytokines, which in turn recruit new immune cells and enhance the immune response [31]. Our previous results showed that supplementation of feed with oat beta-glucans significantly modulated the expression of the genes involved in the signaling pathways of cytokines and their receptors [32]. The present study showed that oat beta-glucans influenced the expression of chemokines and their receptors, which are considered integral components and regulators of inflammation as they serve as chemoattractants, recruiting immune cells to the site of inflammation. Both beta-glucans appear to accelerate remission by influencing the expression of chemokines and their receptors. Significantly increased gene expression of chemokines and their receptors was observed in the animals with TNBS-induced *colitis* at both time points of the experiment. Moreover, the present study showed that oat beta-glucans modulated the gene expression level of chemokines and their receptors. After 3 days of *colitis* induction, increased expression (more than 2-fold change) of 11 genes encoding chemokines (*Ccl2*, *Ccl3*, *Ccl4*, *Ccl7*, *Ccl12*, *Ccl17*, *Ccl19*, *Cxcl1*, *Cxcl2*, *Cxcl6*, and *Cxcl9*) and 9 genes encoding chemokine receptors (*Ccr1*, *Ccr2*, *Ccr3*, *Ccr4*, *Ccr5*, *Ccr8*, *Cxcr2*, *Cxcr3*, and *Cxcr5*) was observed in rats. Similar results were observed after 7 days in the *colitis* group with increased expression of 13 genes encoding chemokines and 6 genes encoding chemokine receptors. It should be noted that this is, to the best of our knowledge, the first study that analyzed the effect of oat beta-glucans with the different molar mass on the broad spectrum of expression of genes encoding chemokines and its receptors.

ANOVA analysis of the results for chemokines and their receptors allowed indicating the exact direction of changes of gene expression in the experimental groups. The induced colon inflammation in rats increased the gene expression of chemokines such as *Ccl3*, *Ccl4*, *Ccl5*, *Cxcl1*, *Cxcl2*, *Cxcl9*, and *Cxcl11* and chemokine receptors such as *Ccr1*, *Ccr2*, *Ccr5*, and *Cxcr2*. In addition, our study showed a significant effect of time on the reduction of the gene expression of *Ccl3*, *Cxcl1*, *Cxcl2*, *Cxcl10*, *Cxcl11*, and *Ccr1* after 7 days of TNBS administration compared to 3 days. Most importantly, the results of the FLD analysis confirmed the results of ANOVA and indicated that after 3 days of the experiment, the gene expression of *Ccl3*, *Ccl4*, *Cxcl1*, *Cxcl2*, *Cxcl9*, *Ccr1*, *Ccr2*, *Ccr5*, *Cxcr2*, *Csf3*, *Osm*, and *Spp1* was higher in the rats from the *colitis* βG− group compared to control groups, while after 7 days, only the gene expression of *Ccl4*, *Ccl5*, *Cxcl1*, *Cxcl2*, *Cxcl11*, *Ccr2*, *Ccr5*, *Cxcr2*, and *Spp1* was higher. The increased expression of proteins and/or genes of these chemokines and their receptors is also observed in patients with UC and CD [8,20,33]. Furthermore, increased gene expression of *Cxcl1* and *Cxcl2* and that of their receptor *Cxcr2* was shown in an animal model by Boshagh et al. [34]. These authors induced inflammation of the large intestine characteristic of UC in rats, which suggests that the animal model of UC is also characterized by increased expression of these two chemokines and their receptor. Moreover, Li et al. [35] showed increased expression of CXCL2 and its receptor CXCR2 in the inflamed colon wall by the administration of TNBS, which reflects an animal model of CD. Some other authors have also demonstrated increased gene expression of *Ccl3, Ccl4*, and *Ccl5* and that of their receptors *Ccr1* and *Ccr5* in animal models of inflammation [36,37,38].

In our study, the consumption of low- or high-molar-mass oat beta-glucans reduced the gene expression of some of the chemokines and their receptors, especially after 3 days of *colitis* induction. After 3 days, the colon inflammation in rats was found to be of acute local type, with a large increase in the gene expression of chemokines and their receptors as well as in the expression of proteins and/or genes of cytokines and their receptors and other markers of inflammation, with slight changes in the blood parameters [32,39]. After 3 days of *colitis* induction, reduced expression of genes such as *Ccl3* and *Ccl4* and that of chemokine receptors *Ccr1* and *Ccr2* was observed in *colitis* animals receiving feed supplemented with low-molar-mass oat beta-glucan, while the animals receiving feed with high-molar-mass beta-glucan showed decreased expression of the chemokines *Ccl3*, *Ccl4*, *Cxcl1*, and *Cxcl2* and the receptors *Ccr1*, *Ccr2*, *Ccr5*, and *Cxcr2*. These results clearly indicate the greatest effect of high-molar-mass oat beta-glucan on the modulation of the gene expression of chemokines and their receptors in the colon wall during the acute phase of inflammation. Xie et al. [40] showed a similar relationship to the gene expression of *Ccl3* chemokine and subfamily of C-X-C chemokine (*Cxcl1*, *Cxcl2*, *Cxcl3*). The authors observed reduced expression of these genes in the rats with DSS (dextran sulfate sodium)-induced *colitis* consuming feed with fungal beta-glucan. They also found an increase in the gene expression of *Ccl5* in rats with inflammation consuming feed with beta-glucan [40].

The chemokines CXCL1 and CXCL2 and their receptor CXCR2 play an important role in experimental *colitis* because the deletion of CXCR2 alleviates chronic *colitis* and associated tumors by inhibiting the penetration of myeloid derived suppressor cells (MDSCs) into the colon mucosa. In inflammation and/or cancer of the colon, the expression of CXCL1 and CXCL2 is increased, which causes the chemotaxis of MDSCs. Moreover, in IBD, the CXCR2 receptor mediates the migration of neutrophils and monocytes to the site of inflammation, resulting in an inflammatory infiltrate. Blocking this receptor can aid in reducing the severity of *colitis* by regulating the function of neutrophils [12]. Thus, the effect of high-molar-mass oat beta-glucan on the reduction of gene expression of *Cxcl1*, *Cxcl2*, and *Cxcr2*, observed in our study, seems to be beneficial. This may be associated with a reduction in the infiltration of the inflamed colon wall by neutrophils and monocytes, which may contribute to reducing inflammation.

The results of protein expression of selected chemokines (CXCL1 and CCL5) and their receptors (CXCR2 and CCR5) observed in our study indicate a complex relationship between gene expression and protein expression at the site of intestinal inflammation. The study showed that the protein expression of CXCL1 was significantly reduced after 3 and 7 days of TNBS-induced *colitis*. However, it should be noted that the protein expression of this chemokine was measured only in the mucosa, while its gene expression was measured in the entire colon wall. The consumption of feed with high- or low-molar-mass oat beta-glucan resulted in an increase in the protein expression of CXCL1, while it decreased the gene expression of this chemokine. However, there are no reports indicating the influence of cereal beta-glucans on the expression of this chemokine in *colitis,* but some data highlight the influence of beta-glucans on other origins. Fungal beta-glucans have a stimulating effect on CXCL1 expression in macrophages that infiltrate the site of inflammation [41]. Moreover, intestinal epithelial cells show very high expression and secretion of CXCL1 [42], as was observed in our study and shown in Figure 4A. The increase in the number of macrophages showing increased secretion of CXCL1 causes an increase in the concentration of this protein at the site of the inflammatory infiltrate, mostly in the submucosal layer, which was not included in the immunohistochemical analysis of the protein expression in this study. Moreover, severe mucosal damage caused by induced *colitis* resulted in low expression of CXCL1 due to damage to the intestinal epithelial cells. Thus, the high gene expression of *Cxcl1* in the inflamed intestinal wall may be associated with a significant infiltration of inflammatory cells (including macrophages) in the submucosa, which also includes endothelial cells and TNF-α-stimulated pericytes to produce CXCL1 [19]. The consumption of oat beta-glucans resulted in a protective effect on the intestinal mucosa, which reduced the damage to the intestinal epithelial cells and resulted in higher secretion of CXCL1, similar to that observed in rats from the control groups.

The direction of changes in the protein expression of CXCR2 is surprising, because this receptor, which is responsible for mediating the migration of neutrophils to the inflammation site, should be high in rats with induced *colitis*. However, our study showed that it was at a very low level in the *colitis*-induced animals at both 3 and 7 days after *colitis* induction, while *colitis* rats fed with feed containing low- or high-molar-mass oat beta-glucans showed increased expression of CXCR2 in the mucosa after 7 days of TNBS administration. This clearly indicates that the expression of this receptor protein has a stimulating effect on the inflamed mucosa. The literature data indicate that increased expression of CXCR2 may intensify and maintain the inflammatory infiltration (of the colon wall by leukocytes, in particular, neutrophils) [12,19]. It is also worth noting that in our study, rats from the *colitis* group consuming feed with beta-glucans showed high protein CXCR2 expression in inflammatory infiltration cells, as well as in intestinal crypt cells, especially near the area of inflammatory infiltrates and heavily damaged mucosa (Figure 4A). Therefore, it can be assumed that along with an increase in the expression of CXCR2 and the ligand of this receptor (CXCL1), the number of neutrophils and monocytes also increased. It is important to focus on the functions of neutrophils and monocytes in the body. Thanks to their strong reactivity to pathogens and their antibacterial activity, neutrophils protect the body against external pathogens, especially during damage to the intestinal barrier. On the other hand, tissue-resident monocytes can be transformed into macrophages, which after a microbial challenge are activated to produce neutrophil chemoattractants such as CXCL1 and CXCL2 [43]. Taking into account the early stage of acute induced inflammation and the role of neutrophils and monocytes/macrophages in the destruction and neutralization of pathogens, it seems that their fast infiltration caused by oat beta-glucans can be beneficial to the organism. This effect is related to a faster and more intense immune response to the damage of the intestinal barrier caused by the rectal administration of TNSB. Our previously published results confirmed the accelerated remission and a milder course of inflammation in rats with *colitis* [32,39,44]. Interestingly, earlier studies have shown that neutrophils may also play a protective role during TNBS- and DSS-induced *colitis* [45,46]. Therefore, the function of neutrophils may result from changes in the inflammatory environment in the colon, which depend on the *colitis* model used and the stage of development of induced inflammation [43].

Modulation of the expression of CXCR2 expression and its ligand CXCL1 by oat beta-glucans is accompanied by the activation of the CXCLs/CXCR2 signaling pathway. Activation of this pathway can induce multiple G protein-mediated signaling cascades, including phosphatidylinositol-3 kinase (PI3K)/Akt, phospholipase C (PLC)/protein kinase C (PKC), and mitogen-activated protein kinase (MAPK)/p38 pathways, as well as Janus kinase (JAK2)/signal transducer and activator of transcription (STAT3) signaling pathway [12]. Activation of these pathways has an antiapoptotic effect and increases cell proliferation, which correlates with the antiapoptotic effect of oat beta-glucans observed in the same animals, especially after 7 days of *colitis* induction [47].

In our study, the induced inflammation caused an increase in the gene expression of *Ccl5* only after 7 days of TNBS administration, while an increase in the protein expression of this chemokine in the mucosa was observed after 3 days. Furthermore, the protein expression of CCL5 decreased after 7 days. The significance in the gene and protein expression of CCL5 changes were also confirmed by the results of the FLD analysis, which clearly confirmed the direction of changes observed after 7 days of *colitis* induction. The rats from the CβG− group showed an increase in the gene expression of *Ccl5* and a decrease in the protein expression of CCL5, while the rats from the CβGh+ group showed no change in the gene expression of *Ccl5* and increased protein expression of CCL5. CCL5 can be produced by many cell types, including platelets, macrophages, T cells, fibroblasts, and endothelial and intestinal epithelial cells [14]. This chemokine is involved in biological functions associated with several physiological and pathological processes, among others, by activating downstream signaling pathways such as STAT3, nuclear factor NF-κB, and MAPK pathway via three receptors on the surface of cells: CCR1, CCR3, and CCR5 [48,49]. Activation of these pathways induces the expression of proinflammatory cytokines such as TNF-α, IL-1β, and IL-6 [50]. Yu et al. [51] showed that platelets play an important proinflammatory role in a mouse model of DSS-induced *colitis*. The authors observed that platelet depletion abolished the DSS-induced increase in the expression of CCL5, which indicates that platelets are an important source of this chemokine in acute *colitis*. Moreover, platelets regulate the infiltration of neutrophils in acute *colitis* through the secretion of CCL5, which stimulates the production of CXC chemokines, including CXCL2, which is a chemoattractant for neutrophils [51]. Moreover, our study showed a significant increase in the number of platelets in the plasma after 3 days of TNBS administration in the rats with induced *colitis* (CβG−) and a significantly lower number in *colitis* rats consuming feed with low-molar-mass oat beta-glucan (CβGl+) [39], which positively correlates with changes in CCL5 expression in these experimental groups. Furthermore, Nieto et al. [52] showed that CCR5/CCL5 modulates the inflammatory response induced by TLR4 ligands in the monocytes involved in the pathogenesis of CD. Blocking the CCR5 receptor decreased the migration potential and the percentage of CD14+CD163+ monocytes, but it increased the release of TNF-α and IL-6 induced by TLR4 ligands. The addition of CCL5 and activation of the CCR5 receptor pathway increased the percentage of CD14+CD163+ monocytes and decreased the production of TNF-α and IL-6 [52]. Thus, the increase in CCL5 observed at 7 days after TNBS administration in *colitis* rats consuming feed with high-molar-mass oat beta-glucan (βGh+) appears to be beneficial.

The high-molar-mass oat beta-glucan significantly suppressed the increase in the gene expression of *Ccr5* caused by the induced *colitis*, while low- and high-molar mass oat beta-glucan caused a reduction in the protein expression of CCR5 after 3 days of TNBS administration in the *colitis* and control groups. It should be noted that the expression of CCR5 was high after 7 days of inflammation in the control group not consuming beta-glucans compared to the rest of the experimental groups, while beta-glucans lowered the expression of this receptor. CCR5 is expressed by various leukocytes and plays a key role in the development of acute and chronic inflammation. Based on an analysis of CCR5 expression on leukocytes in mice with TNBS-induced *colitis*, Mencarelli et al. [53] showed that the development of inflammation in this model is mediated by the recruitment of macrophages, monocytes, and granulocytes [53]. The pathogenesis of CD mainly involved Th1 cells, which also play a key role in the animal models of *colitis* induced using TNBS [29,54,55]. Th1 cells are characterized by high expression of the CCR5 receptor; therefore, the increase in CCR5 expression correlates with the intensity of inflammation. Moreover, this receptor is chemotactic for CD4+ cells, the number of which increases during inflammation [53]. In the active phase of CD, CCR5 is mainly expressed in the membrane and cytoplasm of glandular epithelial cells, vascular endothelial cells, and inflammatory cells in the lamina propria [56]. Immunohistochemical images indicated that TNBS-induced *colitis* increased the expression of CCR5 in the glandular epithelial cells of the colon and in inflammatory cells in the lamina propria, especially after 3 days of induced *colitis* and in highly damaged and inflamed areas (Figure 4A). After 7 days of *colitis* induction, the CCR5 expression in the inflamed tissue was localized deep inside the intestinal crypts and in the cells of the inflammatory infiltrate, which was mainly located in the submucosa due to damage to the intestinal barrier and the possible penetration of pathogens into the deeper layers of the colon wall. It should be noted that only mucosa was included in the immunohistochemical analysis, which may be the reason for the observation of low CCR5 expression after 7 days of the experiment. On the other hand, in the control rats, CCR5 was expressed in the entire thickness of the mucosa, as well as in the intestinal epithelial cells, which are the most exposed to external pathogens.

Our study also showed a significant effect of high-molar-mass oat beta-glucan on the gene expression of three factors that are essential for the pathogenesis of IBD: *Osm, Csf3*, and *Spp1* (encoding OPN). After 3 days of TNBS administration, the expression of these three genes was significantly increased in rats with *colitis*, while in animals consuming feed with high-molar-mass oat beta-glucan (βGh+), it remained at the same level as in the control groups. In addition, immunohistochemical analysis of OPN protein expression showed that the expression of this protein was reduced in the inflamed mucosa of the colon by both oat beta-glucan fractions. As indicated by the literature review by Iida et al. [24], OPN plays an important role in the development of effective immune response of Th1 cells and the antiapoptotic effect on a number of immune cells: macrophages dendritic cells, NK cells, and T and B cells. Most of all, it stimulates the migration, accumulation, and retention of macrophages at the sites of damage and can modulate cytokine production by promoting Th1 cell-dependent immunity [24]. Oz et al. [57] showed a significant increase in plasma OPN concentration in a mouse TNBS-induced *colitis* model, which correlated with an increase in IL-6 concentration. Moreover, OPN-deficient mice exposed to TNBS showed reduced infiltration of macrophages in the lamina propria compared to the wild-type animals [57]. In contrast, studies by other authors indicate a protective effect of OPN against acute *colitis*, but not against chronic *colitis*, in an experimental inflammation model [58,59]. In addition, Tang et al. [60] showed that the level of OPN was decreased in the intestinal epithelial cells in human IBD samples and DSS-induced mouse *colitis*. The authors also indicated that OPN overexpression protects epithelial cells from the cytotoxicity of TNF-α [60]. The role of OPN in IBD may depend on the type of IBD/experimental model and the phase of the disease as well as on the type of cells (whether they are intestinal epithelial cells or immune cells). OSM belongs to the IL-6 cytokine family and is produced mainly by monocytes/macrophages, dendritic cells, and T lymphocytes, and its level positively correlates with the severity of CD and UC [22]. In addition, OSM, through the OSMR receptor, mediates the activation of JAK-STAT, MAPK, and Akt pathways, and thus, its overexpression intensifies the inflammatory changes in IBD [21]. G-CSF is an important factor that stimulates the granulocytopoiesis of neutrophils in the bone marrow, whereby increased CSF3 expression is associated with an increased infiltration of neutrophils to the site of inflammation, which reduces these immune cells in the blood, stimulating an increase in CSF3 and resulting in the production of neutrophils in the bone marrow [20]. Furthermore, CSF3 regulates innate and adaptive immune responses by modulating the activation of macrophages and dendritic cells. Patients with IBD, who often have a defective immune response, show decreased CSF3 expression, which impairs the immune response to pathogens, and consequently, may exacerbate inflammation [61]. In addition, CSF3^−/−^ mice are more susceptible to acute DSS-induced *colitis* than wild-type mice due to an impaired immune response [62]. In our study, the inhibition of increased *Csf3* gene expression by high-molar-mass oat beta-glucan in the acute phase of induced inflammation may be associated not with impaired immune response but with a protective effect against the pathogens present in the lumen of the colon damaged by rectal TNBS administration.

## 4. Materials and Methods

### 4.1. Animals and Experimental Design

**Figure 7:**
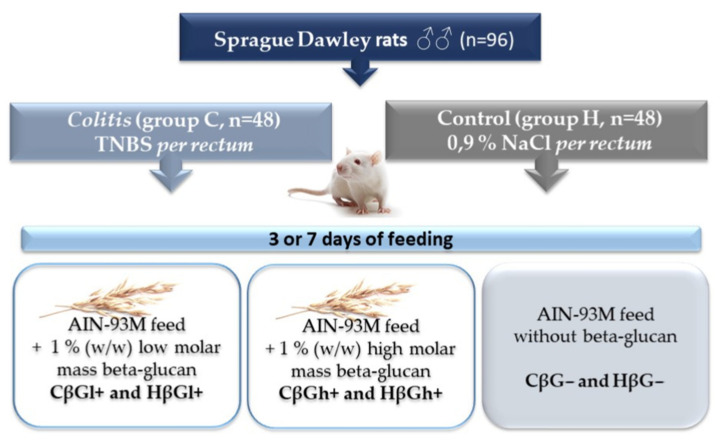
Scheme of the experimental design [32].

The extraction of low- and high-molar-mass oat beta-glucans, as well as the in vivo experiment conducted on rats, has been described in detail in our previous papers [39,44,63]. Briefly, the in vivo experiment involved 96 adult male outbred (CRL:CD(SD) Sprague–Dawley rats (Charles River Laboratories, Sulzfeld, Germany), which were divided into two main groups: control (healthy control—H) and *colitis* (experimentally induced colon inflammation—C). Rats from the *colitis* group were rectally administered with an alcoholic TNBS solution (Sigma Aldrich, Darmstadt, Germany), while rats from the control group were rectally administered a 0.9% saline solution. After the administration of TNBS or saline solution, the rats from each of the two main groups were further divided into three dietary subgroups: group fed with AIN-93M feed containing 1% (*w*/*w*) low molar mass oat beta-glucans (CβGl+ group and HβGl+ group), group fed with AIN-93M feed containing 1% (*w*/*w*) high-molar-mass oat beta-glucans (CβGh+ group and HβGh+ group), and group fed with AIN-93M feed without oat beta-glucans (CβG− group and HβG− group). The animals were fed with feed ad libitum for 3 or 7 days and then were bled from the heart in deep isoflurane anesthesia (Figure 7).

### 4.2. RNA Isolation, Reverse Transcription, and Real-Time PCR

RNA isolation, reverse transcription, and real-time polymerase chain reaction (RT-PCR) were performed as described by Żyła et al. [44]. Briefly, the total RNA was isolated from the colon samples using the RNeasy Lipid Tissue Mini Kit (Qiagen, Hilden, Germany) according to the manufacturer’s instruction. The concentration and purity of the isolated RNA were determined using NanoDrop™ 2000 spectrophotometer (Thermo Fisher Scientific, Waltham, MA, USA). The RNA integrity was assessed using an Agilent Bioanalyzer 2100 system with RNA 6000 Nano LabChip^®^ kit (Agilent Technologies, Palo Alto, CA, USA), assuming the minimal acceptable RNA integrity as 9. Then, RNA was converted to complementary DNA using RT2 First Strand Kit (Qiagen, Hilden, Germany) and prepared for RT-PCR assay as per the manufacturer’s instruction. The expression profiling was performed using RT² Profiler™ PCR Rat Inflammatory Cytokines and Receptors array (Qiagen, Hilden, Germany) based on the manufacturer’s instruction in one technical replicate for each colon sample. PCRs were carried out on Stratagene Mx3005P qPCR system (Agilent Technologies, Palo Alto, CA, USA), with an initial 10-min step at 95 °C followed by 40 cycles of 95 °C for 15 s and 60 °C for 1 min. Relative gene expression was calculated using the ΔΔCt method with Ldha and Rplp1 as housekeeping genes in Data Analysis Qiagen Center (Qiagen, Hilden, Germany). The results are expressed as the relative gene expression of the target vs. reference gene in relation to the healthy control group (HβG−) calculated as 1.

### 4.3. Immunohistochemical Analysis

Large intestine sections with a 5 μm thickness were deparaffinized in xylene and rehydrated in a series of decreasing concentrations of ethanol. Subsequently, the samples were boiled in a microwave twice for 5 min in citrate buffer (pH 6.0) to recover antigens and then incubated in the Bloxall Blocking Solution (Vector Laboratories, Burlingame, CA, USA) to block endogenous enzymes. Next, the samples were incubated at room temperature in 2.5% Normal Horse Serum (Vector Laboratories, Burlingame, CA, USA) and then treated with the following primary antibodies (diluted in 2.5% Normal Horse Serum): rabbit anti-CCL5 antibody (1:1000 dilution; Invitrogen, Carlsbad, CA, USA), rabbit anti-CCR5 antibody (1:1000 dilution; Novus Biologicals, Centennial, CO, USA), rabbit anti-CXCL1 antibody (1:250 dilution; Novus Biologicals, Centennial, CO, USA), rabbit anti-CXCR2 antibody (1:800 dilution; Novus Biologicals, Centennial, CO, USA), and mouse anti-OPN antibody (1:200 dilution; Novus Biologicals, Centennial, CO, USA). The slides with primary antibody were incubated overnight at +4 °C and then washed and labeled with polymers consisting of secondary anti-rabbit or anti-mouse antibodies conjugated to horseradish peroxidase or alkaline phosphatase enzyme complex (Vector Laboratories, Burlingame, CA, USA). The slides were stained with 3,3′-diaminobenzidine (for obtaining brown color) or ImmPACT Vector Red Substrate (for obtaining red color) and then with hematoxylin for nuclei counterstaining. Finally, the samples were dehydrated and preserved by sticking coverslip.

### 4.4. Image Analysis

The immunohistochemical preparations were observed under a ×20 objective lens in a NIKON Eclipse Ti2 microscope. In the recorded photos, six areas of mucosa were marked, and the colorimetric saturation (brown or red color reflecting antigen expression) and object area were measured by using NIS-Elements BR 5.01 program. Then, integrated optical density (IOD) was calculated using the following formula:

$$\mathrm{Integrated}\;\mathrm{Optical}\;\mathrm{Density}\;\left(\mathrm{IOD}\right)=\frac{Object\;area}{Measured\;area}\times Mean\;saturation.$$

### 4.5. Statistical Analysis

The obtained data were analyzed using Statistica software (version 13.3 PL; StatSoft, Kraków, Poland). Prior to the analysis, the normality of distribution and equality of variance were determined for all data by Shapiro–Wilk and Brown–Forsythe tests. To be able to perform statistical analysis, the following data were transformed to obtain the normal distribution and equal variance by natural logarithm (gene expression of *Ccl3, Cxcl1, Cxcl2, Cxcl9, Cxcl10, Cxcl11, Ccr1, Cxcr2*, and *Csf3*), decimal logarithm (gene expression of *Spp1*), and square root (gene expression of *Ccr2, Ccr5*, and *Osm* and immunohistochemical results of CCR5, CXCR2, CXCL1, and OPN). Three-way analysis of variance (ANOVA) was performed to evaluate the influence of three experimental factors (colon inflammation, dietary intervention, period of its use) and the effect of interaction between these factors. The significance of differences between the groups was determined by the Tukey post hoc test. The results of all nutritional subgroups were compared to that of the control subgroup (HβG−) using the Dunnett post hoc test for each time point separately. A difference was considered significant if the *p*-value was below 0.05. The time-dependent effect between the experimental factors was assessed by Fisher’s linear discriminant (FLD) analysis using R statistics software (version 3.3.3; www.r-project.org/, accessed on 5 December 2021; R: The R Project for Statistical Computing).

## 5. Conclusions

In conclusion, our results revealed the important role of oat beta-glucans in inflammation response modulation in inflammatory bowel disease through the influence on chemokines and their receptors signaling pathway. The present study described for the first time the effect of oat beta-glucans of varying molar masses on the gene expression of chemokines and their receptors, and the protein expression of selected chemokines and their receptors, in the inflammation-induced colon, reflecting acute and pre-remission inflammation as a model of human CD. In addition, our study analyzed the influence of beta-glucans on the gene expression of important proteins involved in the pathogenesis of IBD. The results of this study, as well as that of the previously published works, confirm the immunomodulatory properties of both oat beta-glucan fractions [32,44,47]. However, high-molar-mass oat beta-glucan showed a stronger effect on the analyzed parameters and decreased the gene expression of *Ccl3*, *Ccl4*, *Cxcl1*, *Cxcl2*, *Ccr1*, *Ccr2*, *Ccr5*, *Cxcr2*, *Osm*, *Csf3*, and *Spp1* in acute inflammation of the colon wall, and the gene expression of *Ccl5*, *Cxcl1*, *Cxcl2*, *Cxcl11*, *Ccr2*, *Ccr5*, *Cxcr2*, and *Spp1* in the preremission phase (Table 1). Furthermore, this polysaccharide modulated the expression of CXCL1, CXCL5, CXCR2, CCR5, and OPN proteins in the inflammatory colon mucosa, accelerating inflammation suppression. The activity of high-molar-mass oat beta-glucan may be associated with the formation of a physical barrier against pathogens in the damaged and inflamed mucosa of the colon. Our study, to the best of our knowledge, is the first comprehensive report on the effect of *colitis* itself and the modulatory effect of oat beta-glucans of various molar mass consumption on the expression of chemokines and their receptors as well as other colon inflammation-related proteins such as OSM, CSF3, and OPN (coded by the *Spp1* gene) in an experimental model of TNBS-induced IBD.

## Figures and Tables

**Table 1:** Summary table of the direction of changes in the gene expression of chemokines and their receptors as well as other colon inflammation-related proteins in rats with TNBS-induced *colitis* fed with low- or high-molar-mass oat beta-glucans.

		CβG− vs. HβG−	CβGl+ vs. CβG−	CβGh+ vs. CβG−	HβGl+ vs. HβG−	HβGh+ vs. HβG−
3 days	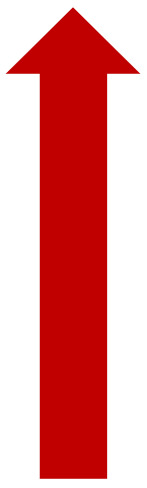	*Ccl3, Ccl4, Cxcl1, Cxcl2, Cxcl9, Ccr1, Ccr2, Ccr5, Cxcr2, Osm, Csf3, Spp1*	*Cxcl11,* CXCL1	CXCL1		
	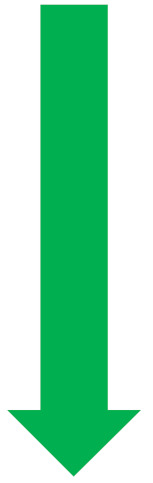	CXCL1	*Ccl3, Ccl4, Ccr1, Ccr2,* CCL5, CCR5, OPN	*Ccl3, Ccl4, Cxcl1, Cxcl2, Ccr1, Ccr2, Ccr5, Cxcr2, Osm, Csf3, Spp1,* CCL5, CCR5, OPN	CXCL1, CCL5, CCR5, OPN	CCR5
7 days	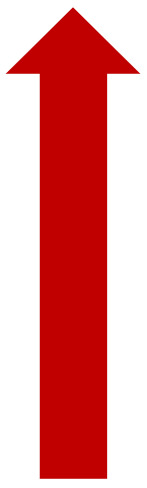	*Ccl5, Cxcl1, Cxcl2, Cxcl9, Cxcl11, Ccr2, Ccr5, Cxcr2, Spp1,* OPN	CXCL1, CXCR2	CXCL1, CCL5, CXCR2	*Cxcl9*	
	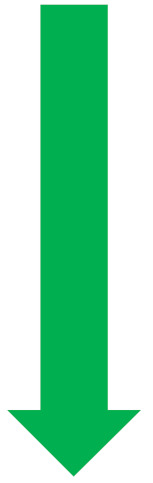	CXCL1, CCR5,	OPN	*Ccl5, Cxcl1, Cxcl2, Cxcl11, Ccr2, Ccr5, Cxcr2,* OPN	CCR5	CCR5

Italic letters indicate genes expression in the colon wall. Not italic and capital letters indicate protein expression in the colon mucosa.

## Data Availability

The data that support the findings of this study are available on request from the corresponding author [K.D.].

## Supplementary Materials

The following are available online at https://www.mdpi.com/article/10.3390/ijms23031406/s1.
