# Supplementary material for: Colon Expression of Chemokines and Their Receptors Depending on the Stage of Colitis and Oat Beta-Glucan Dietary Intervention—Crohn’s Disease Model Study
Source: Int J Mol Sci. 2022 Jan 26;23(3):1406. doi: 10.3390/ijms23031406 (PMC8835763; doi:10.3390/ijms23031406)
Supplement: Supplementary file 1 [file ijms-23-01406-s001.zip › ijms-1559785-supplementary.pdf]

Supplementary figures

The gene expression of chemokines

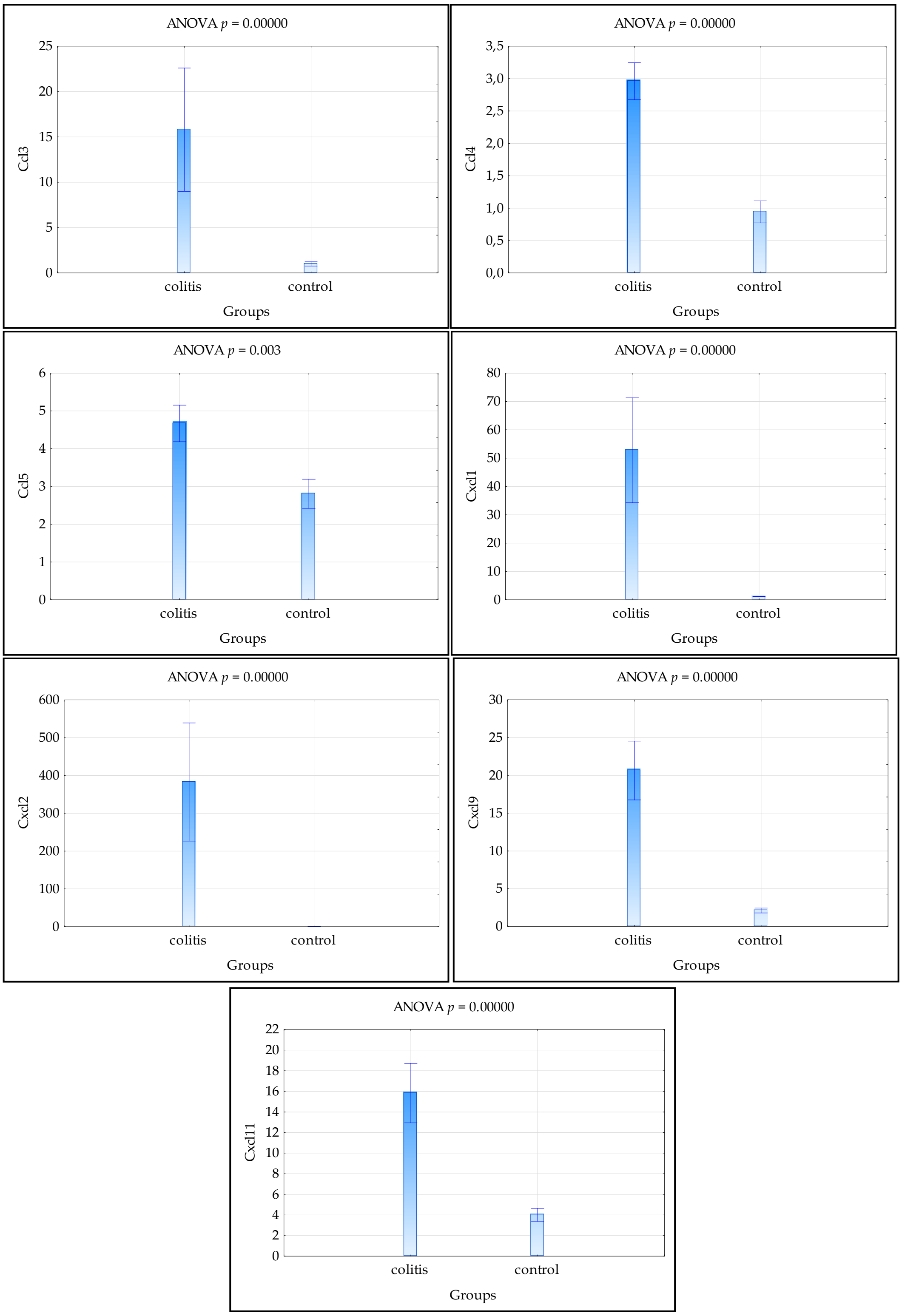

Figure S1. Changes of gene expression of selected chemokines (mean ± SE) by influence of inflammation. ANOVA analysis.

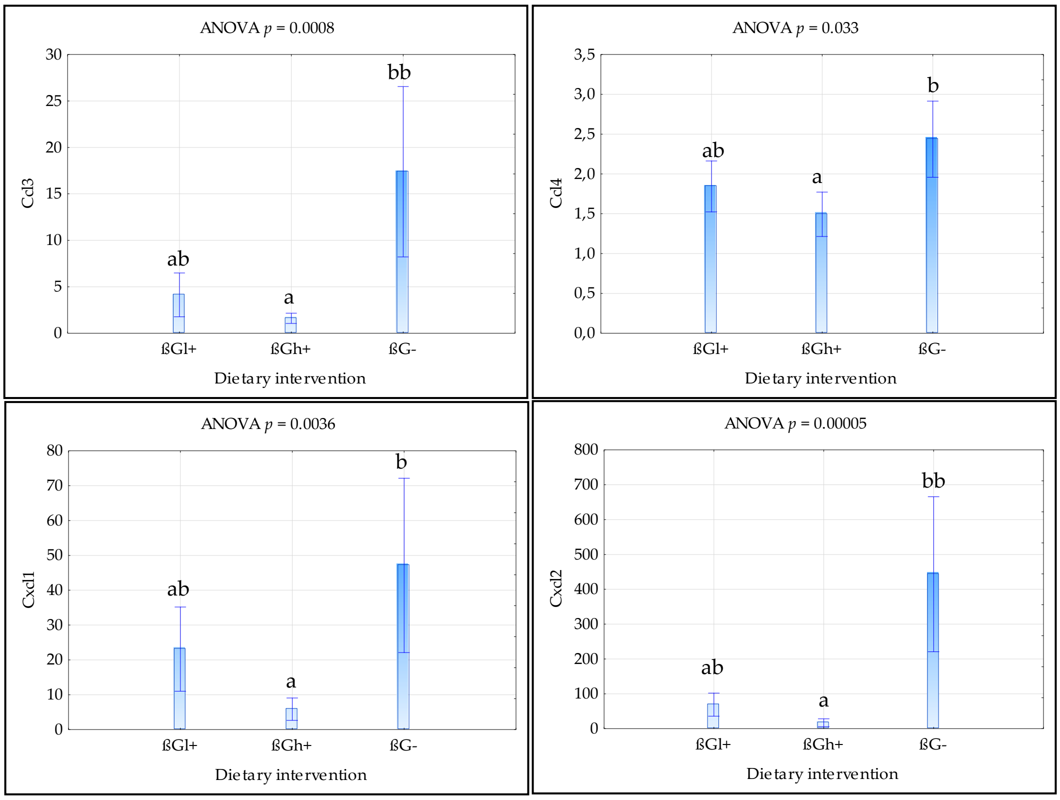

Figure S2. Changes of gene expression of selected chemokines (mean ± SE) by influence of dietary intervention. ANOVA analysis. [a,b] Different letters denote significant differences between dietary group according to the Tukey post-hoc test (a,b $p < 0.05$, a,bb $p < 0.01$).

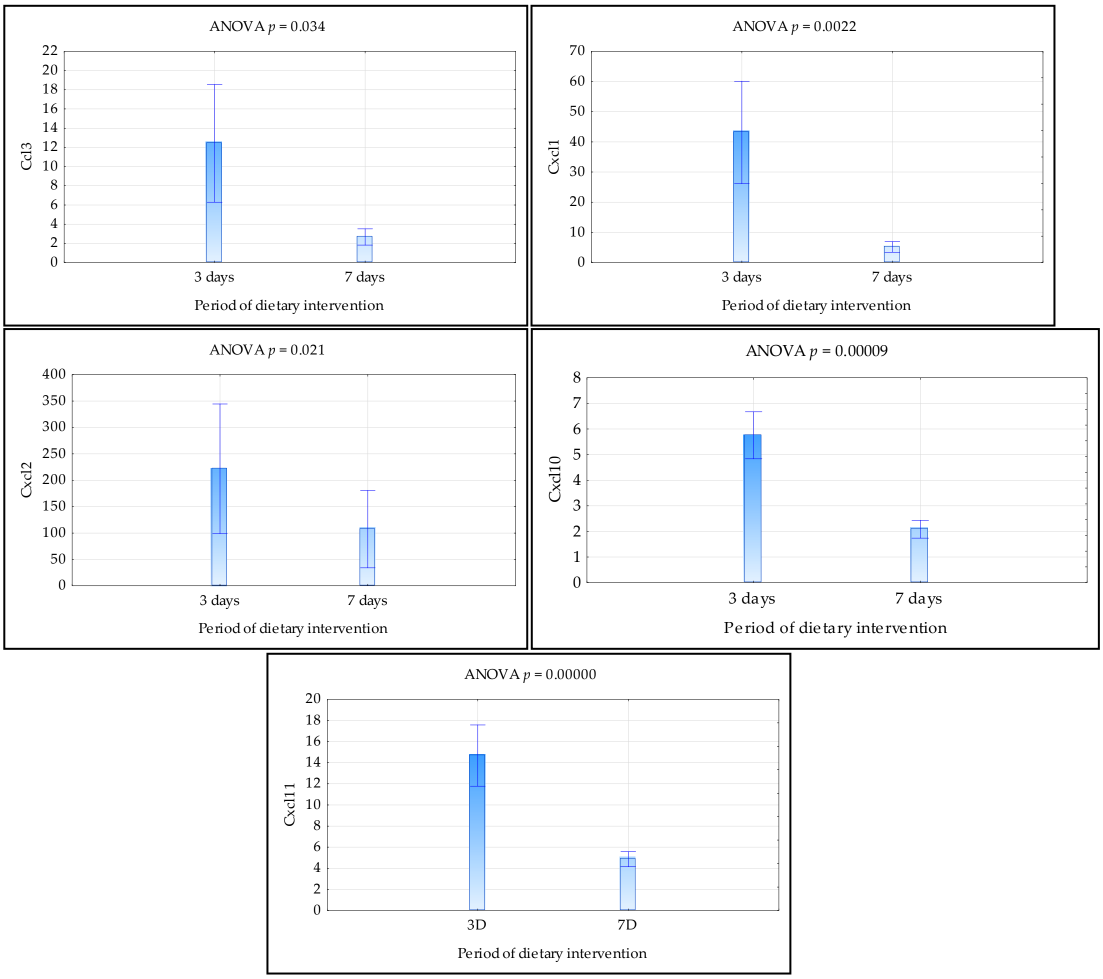

Figure S3. Changes of gene expression of selected chemokines (mean ± SE) by influence of the period of dietary intervention. ANOVA analysis.

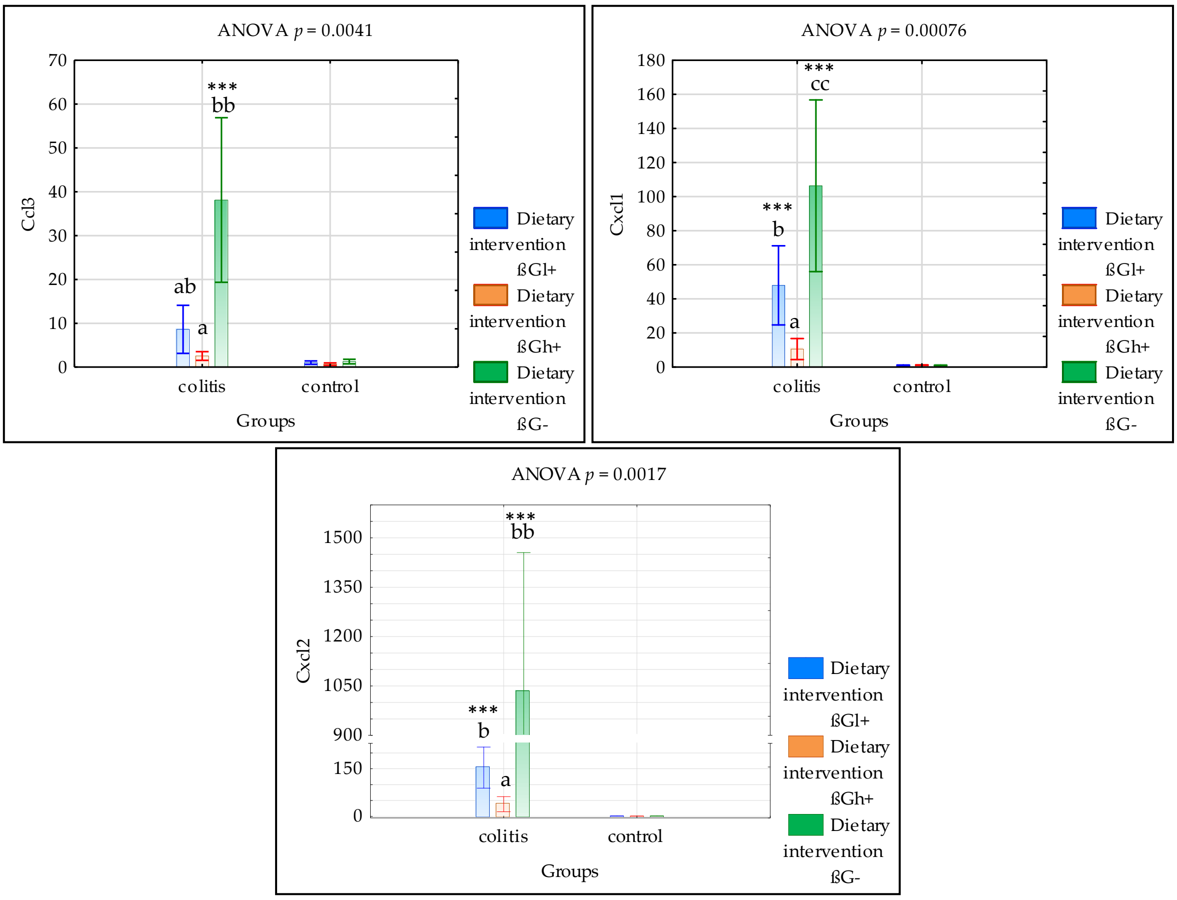

Figure S4. Changes of gene expression of selected chemokines (mean ± SE) by influence of interaction between the period of inflammation and dietary intervention. ANOVA analysis. [a,b] Different letters denote significant differences between dietary groups in the *colitis* group according to the Tukey post-hoc test (a,b p < 0.05, a,bb/cc p < 0.001). * Significantly different from control group (control βG-) according to the Tukey post-hoc test (*** p < 0.001).

The gene expression of chemokine receptors

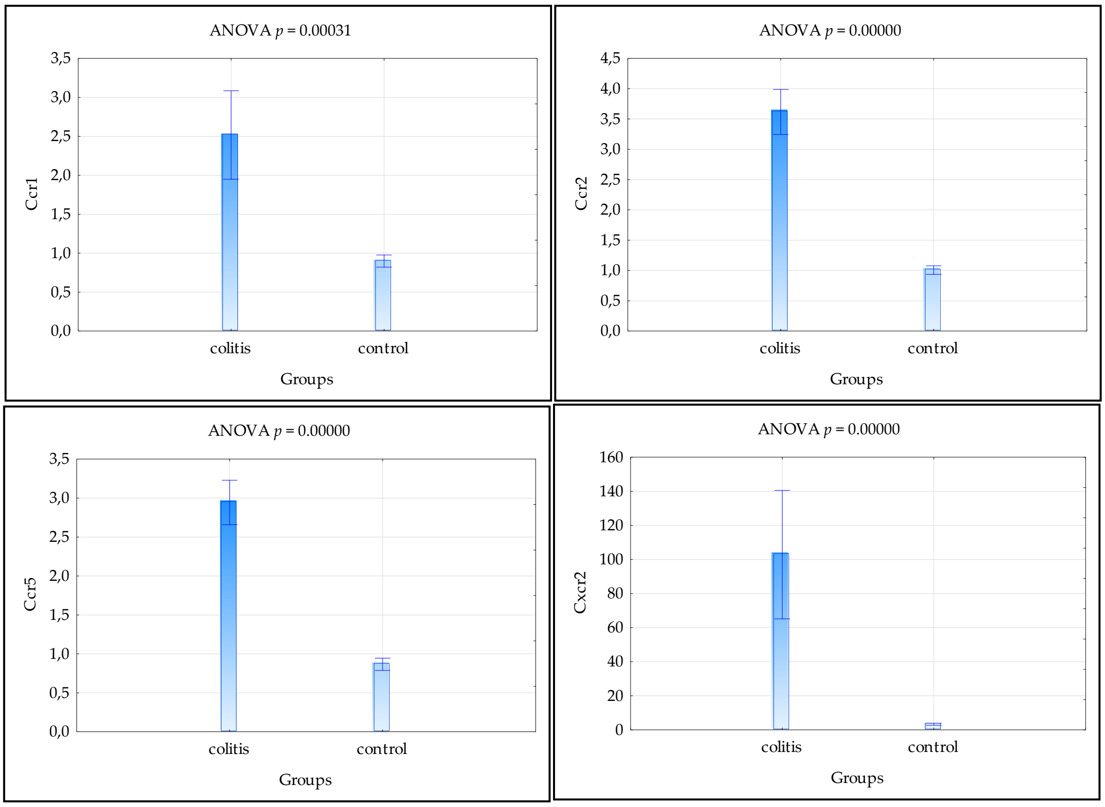

Figure S5. Changes of gene expression of selected chemokine receptors (mean ± SE) by influence of inflammation. ANOVA analysis.

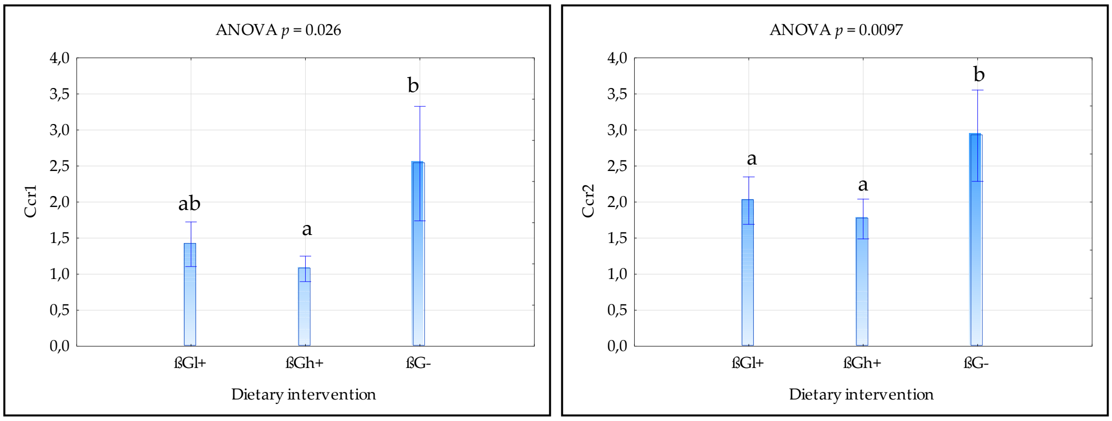

Figure S6. Changes of gene expression of selected chemokine receptors (mean ± SE) by influence of dietary intervention. ANOVA analysis. [a,b] Different letters denote significant differences between dietary group according to the Tukey post-hoc test (a,b $p < 0.05$).

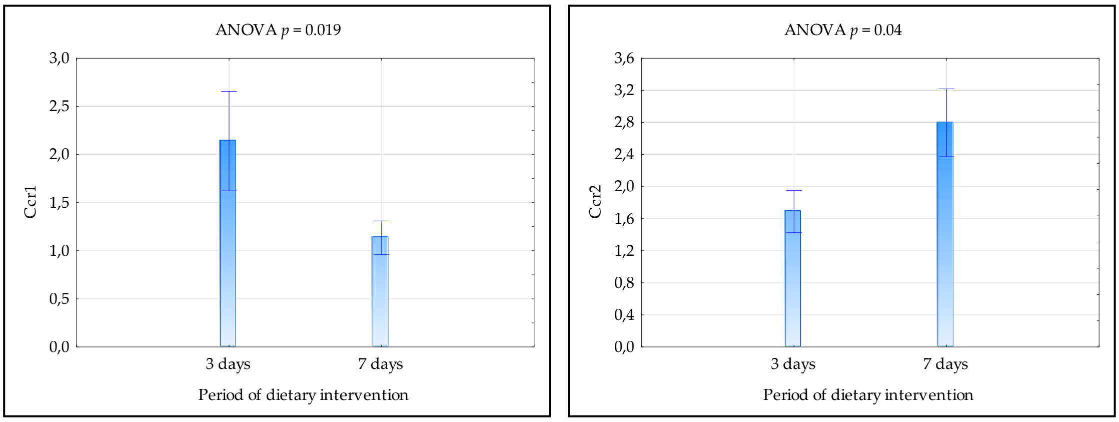

Figure S7. Changes of gene expression of selected chemokine receptors (mean ± SE) by influence of the period of dietary intervention. ANOVA analysis.

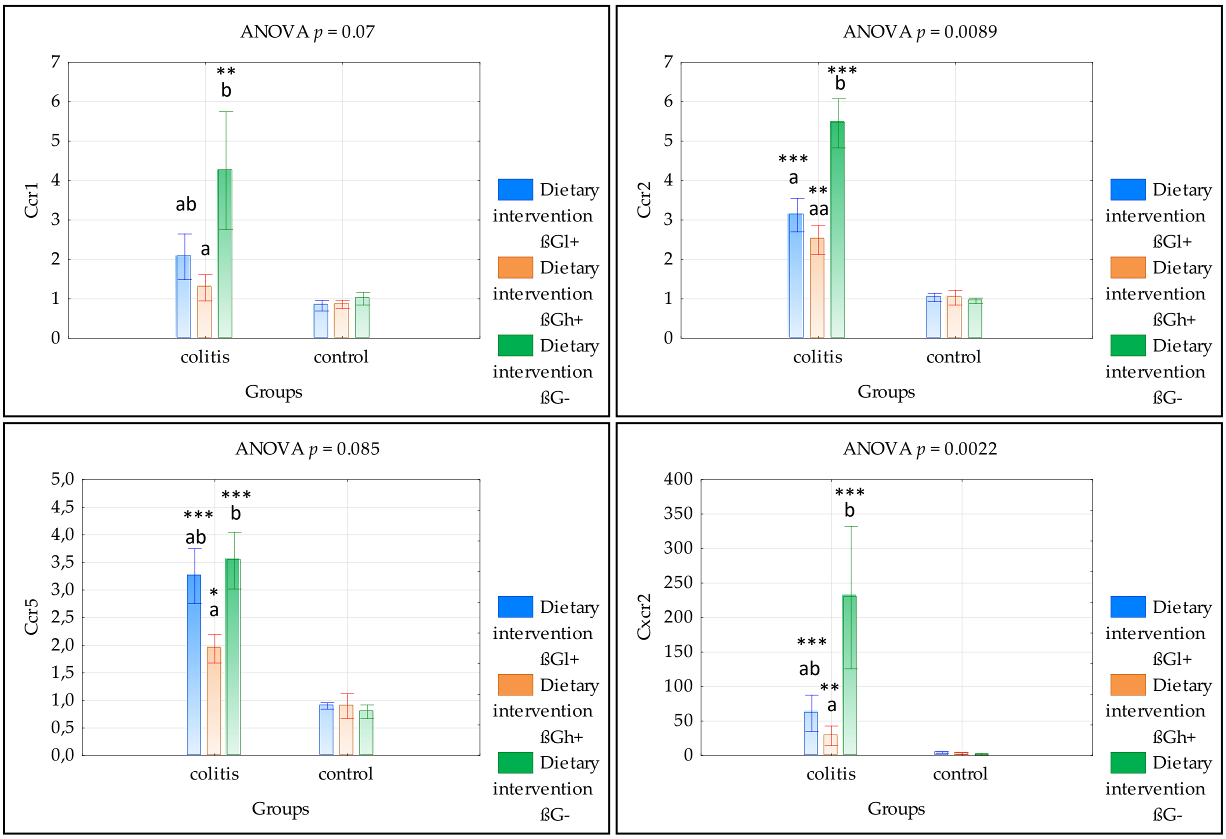

Figure S8. Changes of gene expression of selected chemokine receptors (mean ± SE) by influence of interaction between the period of inflammation and dietary intervention. ANOVA analysis. [a,b] Different letters denote significant between

dietary groups in the *colitis* group according to the Tukey post-hoc test (a,b $p < 0.05$, a,bb/cc $p < 0.001$). * Significantly different from control group (control βG-) according to the Tukey post-hoc test (* $p < 0.05$, ** $p < 0.01$, *** $p < 0.001$).

Osm, *Csf3* and *Spp1* gene expression

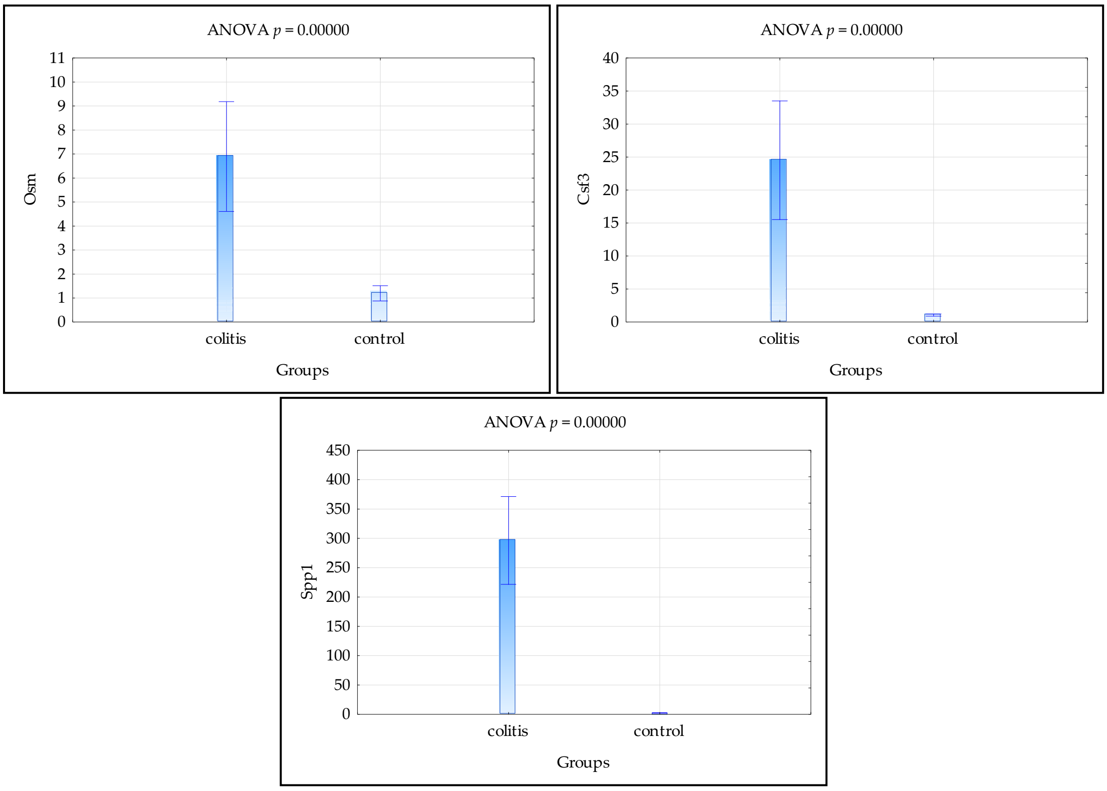

Figure S9. Changes of gene expression of *Osm*, *Csf3* and *Spp1* (mean ± SE) by influence of inflammation. ANOVA analysis.

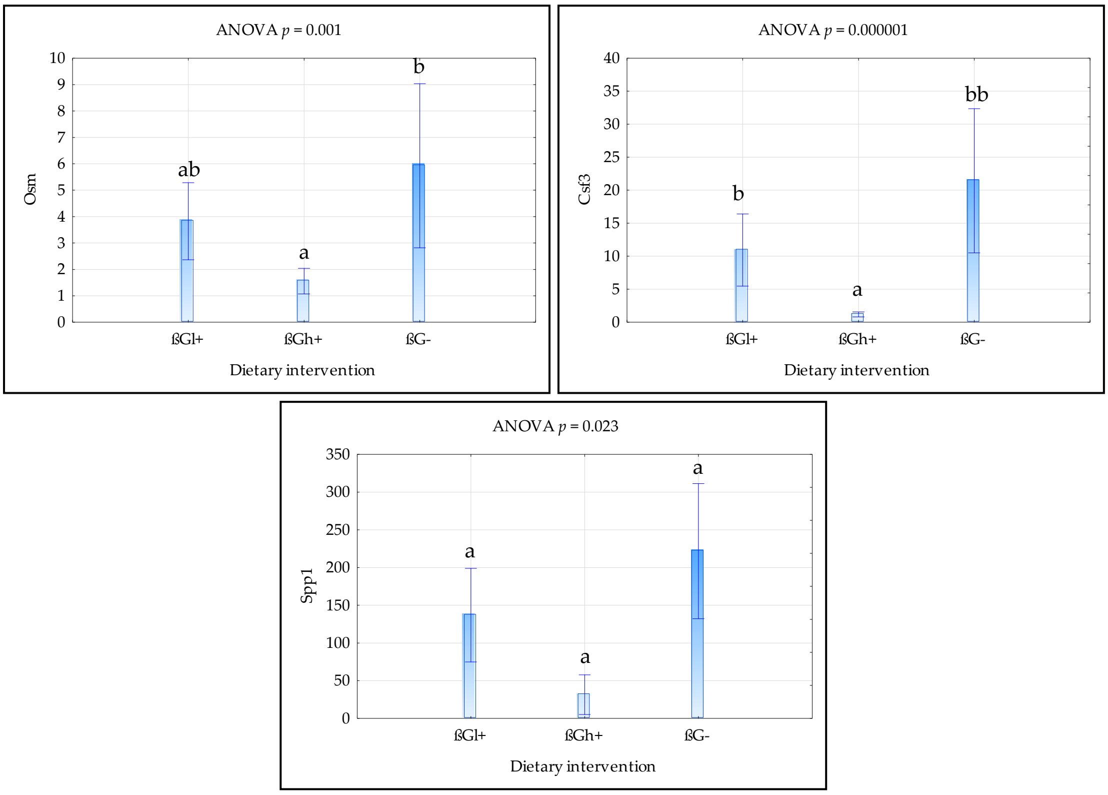

Figure S10. Changes of gene expression of *Osm*, *Csf3* and *Spp1* (mean ± SE) by influence of dietary intervention. ANOVA analysis. [a,b] Different letters denote significant differences between dietary group according to the Tukey post-hoc test (a,b $p < 0.05$, a,bb $p < 0.01$).

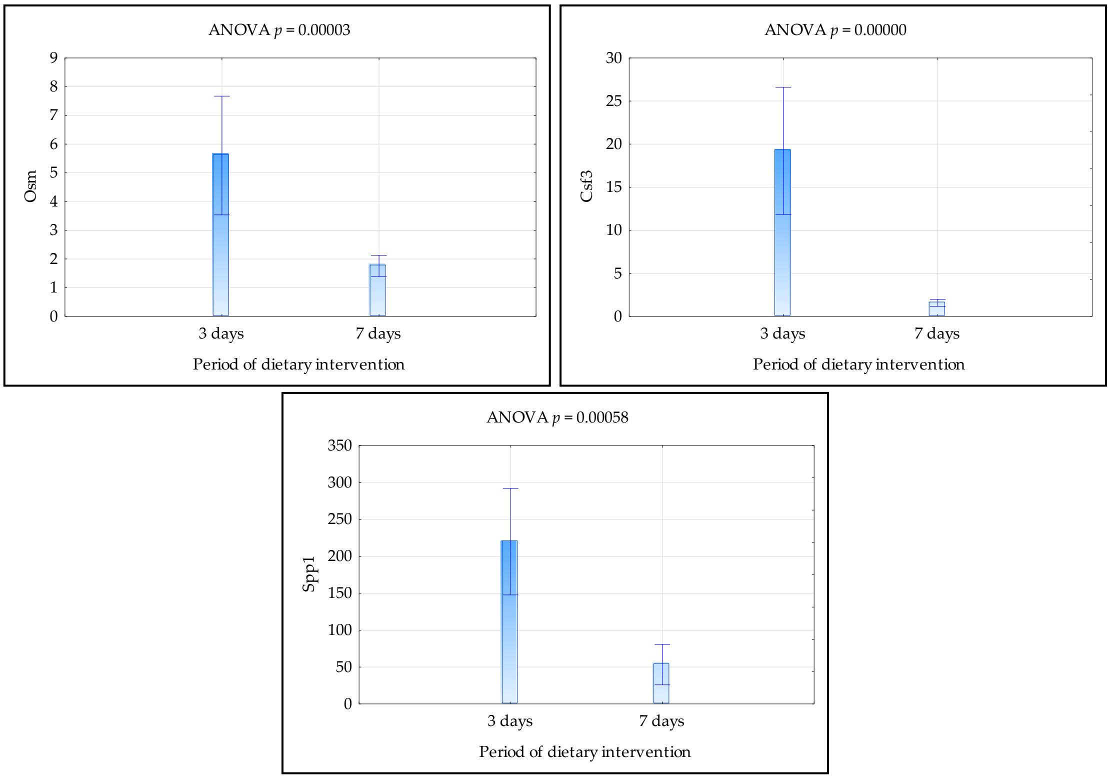

Figure S11. Changes of gene expression of *Osm*, *Csf3* and *Spp1* (mean ± SE) by influence of the period of dietary intervention. ANOVA analysis.

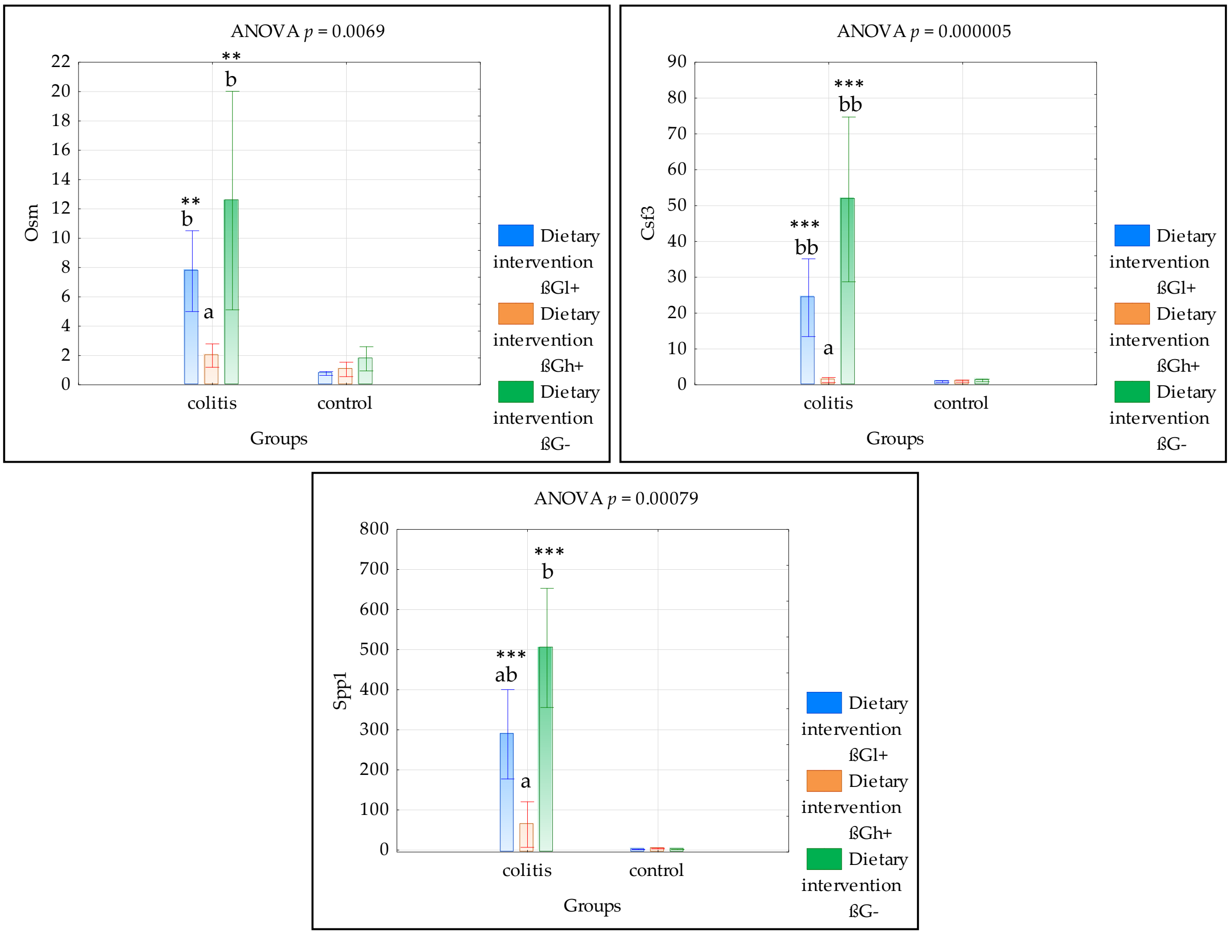

Figure S12. Changes of gene expression of *Osm*, *Csf3* and *Spp1* (mean ± SE) by influence of interaction between the period of inflammation and dietary intervention. ANOVA analysis. [a,b] Different letters denote significant between dietary groups in the *colitis* group according to the Tukey post-hoc test (a,b $p < 0.01$, a,bb $p < 0.001$). * Significantly different from control group (control βG-) according to the Tukey post-hoc test (** $p < 0.01$, *** $p < 0.001$).

Chemokines and their receptors expression in the colon mucosa

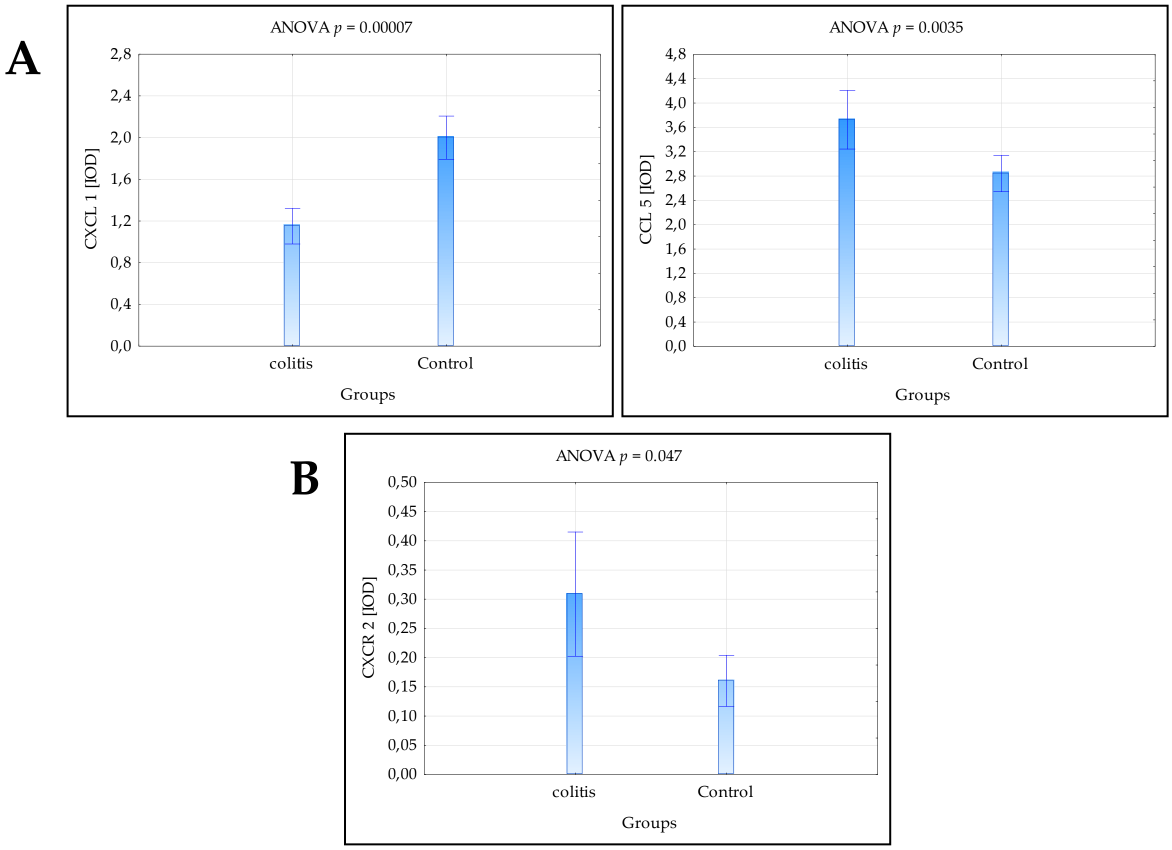

Figure S13. Changes expression of CXCL1 and CCL5 chemokines (**A**) and CXCR2 receptor (**B**) (mean ± SE) by influence of inflammation. ANOVA analysis

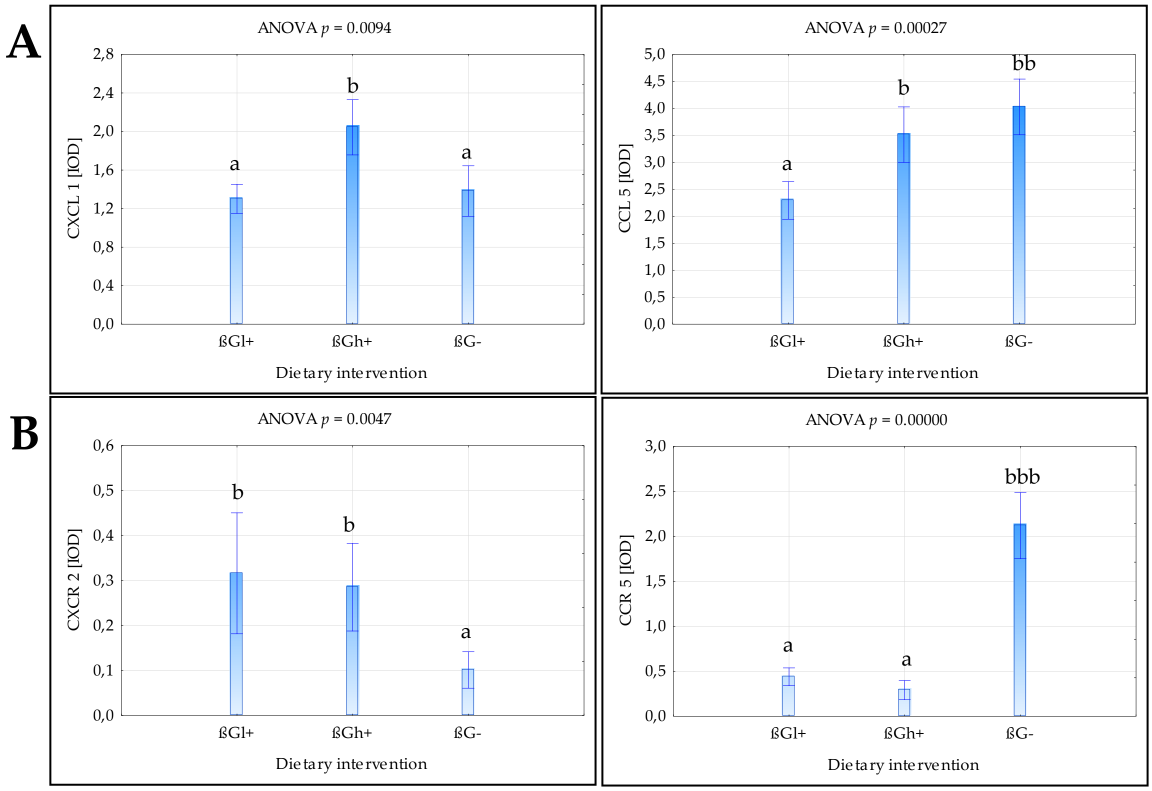

Figure S14. Changes expression of CXCL1 and CCL5 chemokines (**A**) and CXCR2 and CCR5 receptors (**B**) (mean ± SE), by influence of dietary intervention. ANOVA analysis. [a,b] Different letters denote significant differences between dietary group according to the Tukey post-hoc test (a,b $p < 0.05$, a,bb $p < 0.01$, a,bbb $p < 0.001$).

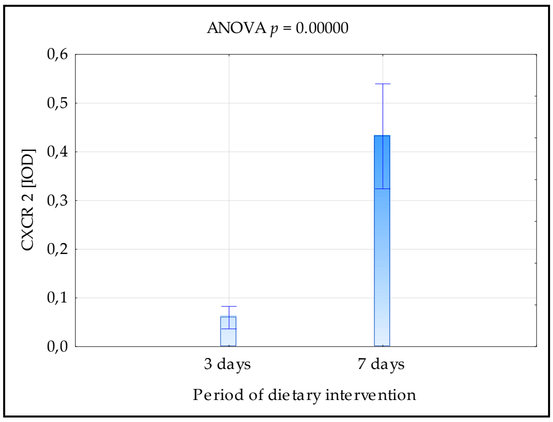

Figure S15. Changes expression of CXCR2 receptor (mean ± SE) by influence of the period of dietary intervention. ANOVA analysis.

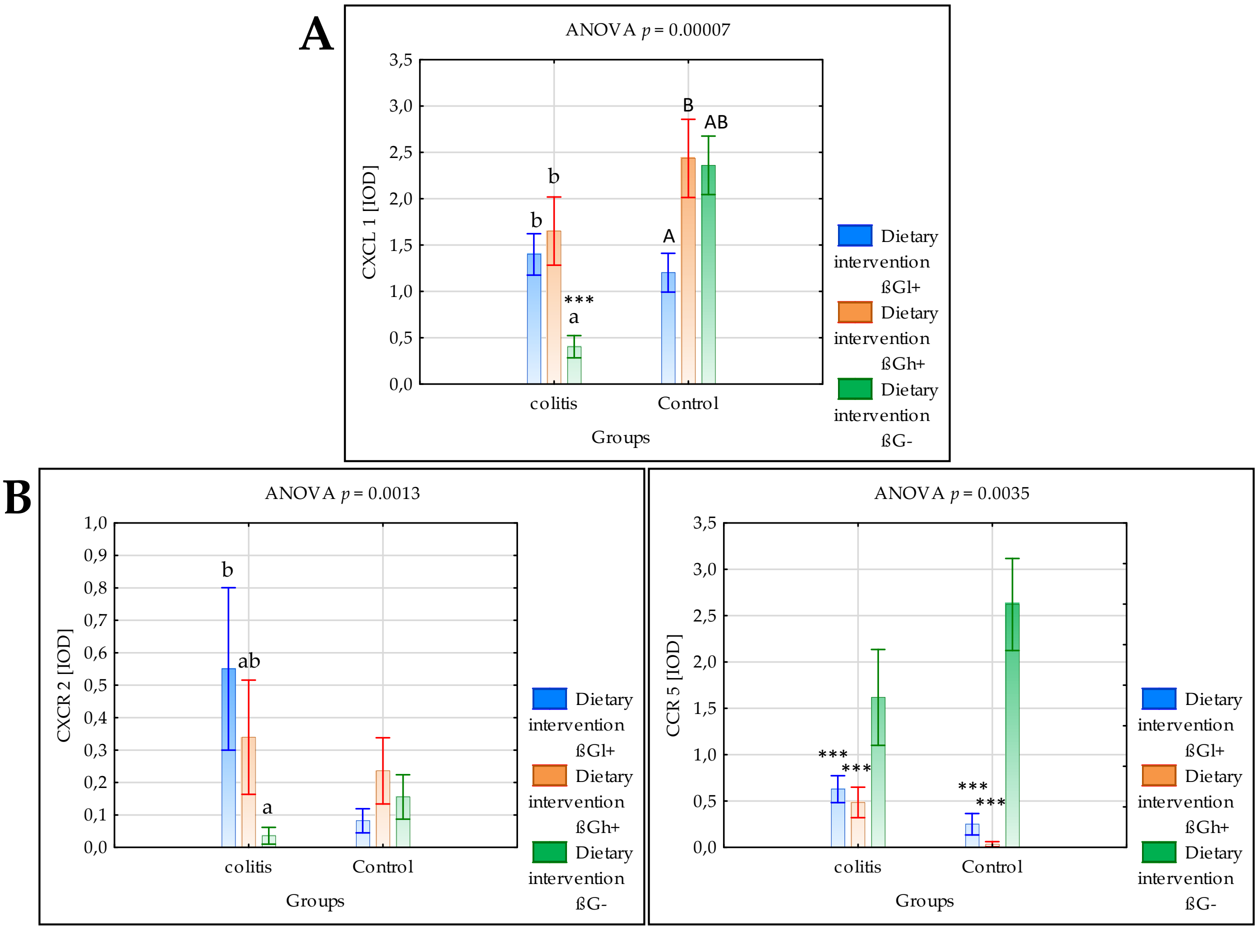

Figure S16. Changes expression of CXCL1 chemokine (**A**) and CXCR2 and CCR5 receptors (**B**) (mean ± SE) by influence of interaction between the period of inflammation and dietary intervention. ANOVA analysis. [a,b] Different letters denote significant between dietary groups in the *colitis* group according to the Tukey post-hoc test (a,b $p < 0.01$). [A,B] Different large letters denote significant between dietary groups in the control group according to the Tukey post-hoc test (A,B $p < 0.05$). * Significantly different from control group (control βG-) according to the Tukey post-hoc test (*** $p < 0.001$).

Osteopontin (OPN) expression in the colon mucosa

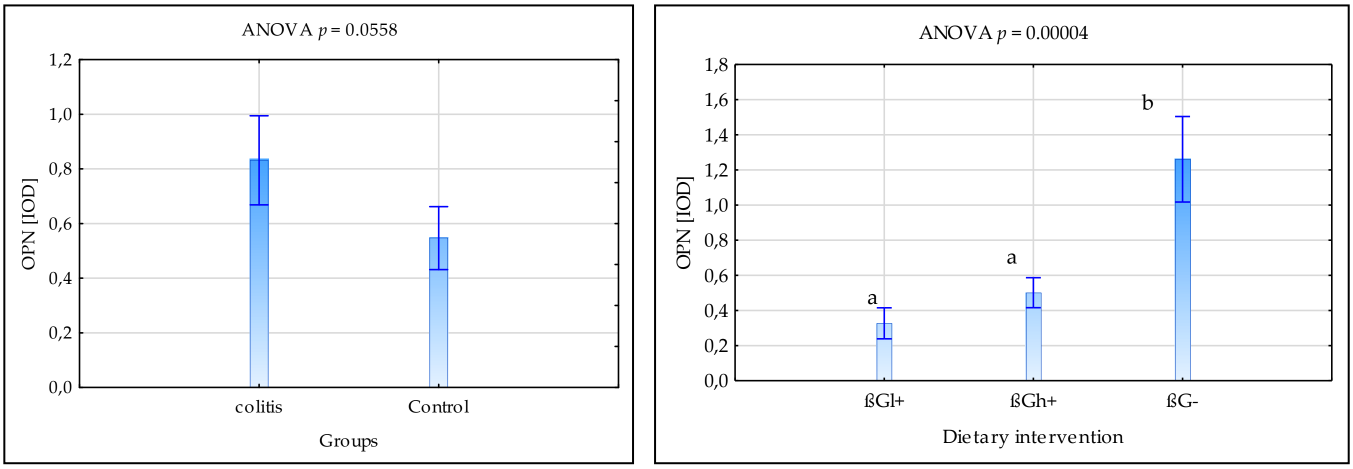

Figure S17. Changes expression of OPN (mean ± SE) by influence of inflammation (**A**) and influence of dietary intervention (**B**). ANOVA analysis. [a,b] Different letters denote significant differences between dietary group according to the Tukey post-hoc test (a,b $p < 0.01$).

		CβG- vs HβG-	CβGl+ vs CβG-	CβGh+ vs CβG-	HβGl+ vs HβG-	HβGh+ vs HβG-
3 days	up-regulation	**Σ 23 gens**<br>CCL and CCR: *Ccl2, Ccl3, Ccl4, Ccl7, Ccl12, Ccl17, Ccl19, Cxcl1, Cxcl2, Cxcl6, Cxcl9, Ccr1, Ccr2, Ccr3, Ccr4, Ccr5, Ccr8, Cxcr2, Cxcr3, Cxcr5*<br>Other: *Csf3, Osm, Spp1*	**Σ 3 gens**<br>CCL: *Cccl10, Cxcl11, Cxcl9*	**Σ 3 gens**<br>CCL: *Ccl20, Ccl24, Cx3cl1*	**Σ 4 gens**<br>CCL and CCR:*Cx3cl1, Ccr8, Cxcr3, Cxcr5,*	**Σ 8 gens**<br>CCL and CCR: *Ccl4, Ccl19, Ccl20, Cx3cl1, Ccr4, Ccr8, Cxcr3, Cxcr5*
3 days	down-regulation	**Σ 2 gens**<br>CCL and CCR: *Cx3cl1*, Ccr10	**Σ 15 gens**<br>CCL and CCR: *Ccl3, Ccl4, Ccl19, Ccl22, Cxcl2, Ccr1, Ccr4, Ccr6, Ccr8, Cxcr1, Cxcr2, Cxcr3, Cxcr5*<br>Other: *Csf3, Osm*	**Σ 16 gens**<br>CCL and CCR: *Ccl2, Ccl3, Ccl4, Ccl7, Ccl19, Cxcl1, Cxcl2, Cxcl6, Ccr1, Ccr2, Ccr5, Cxcr1, Cxcr2*<br>Other: *Csf3, Osm, Spp1*		
7days	up-regulation	**Σ 22 gens**<br>CCL and CCR:*Ccl2, Ccl3, Ccl4, Ccl5, Ccl6, Ccl7, Ccl12, Ccl17, Cxcl1, Cxcl2, Cxcl6, Cxcl9, Cxcl11, Ccr1, Ccr2, Ccr3, Ccr5, Ccr8, Cxcr2*<br>Other: *Csf3, Osm, Spp1*	**Σ 4 gens**<br>CCL and CCR: *Ccl19, Cxcl10, Cx3cr1, Cxcr5*	**Σ 4 gens**<br>CCL and CCR: *Ccl19, Cxcl9, Ccr4, Ccr8*	**Σ 6 gens**<br>CCL and CCR: *Ccl5, Ccl12, Ccl17, Cxcl9, Cxcl11, Cxcr2*	Σ 3 gens<br>CCL and CCR: *Ccl5, Cxcl11, Cxcr2*
7days	down-regulation	**Σ 4 gens**<br>CCL and CCR : *Ccl19, Ccl20, Cx3cl1, Cxcr5*	**Σ 6 gens**<br>CCL: *Ccl20, Cxcl1, Cxcl2*<br>Other: *Csf3, Osm, Spp1*	**Σ 7 gens**<br>CCL and CCR : *Ccl3, Cxcl1, Cxcl2, Cxcr2*<br>Other: *Csf3, Osm, Spp1*	**Σ 4 gens**<br>CCL and CCR: *Cx3cl1, Ccr1, Ccr2, Cx3cr1*	**Σ 12 gens**<br>CCL and CCR: *Ccl3, Ccl9, Ccl19, Ccl20, Cxcl2, Cxcl6, Ccr4, Ccr6, Ccr8, Cx3cr1, Cxcr5*<br>Other: *Osm*

Table S1. Changes in gene expression 3 and 7 days after TNBS administration. Regulation of genes encode chemokines and their receptors and other colon inflammation-related proteins in the colon tissue. Results are reported as fold regulation > 2.

Chemokines and their receptors: chemokine (C-C motif) ligand 2 (*Ccl2*), chemokine (C-C motif) ligand 3 (*Ccl3*), chemokine (C-C motif) ligand 4 (*Ccl4*), chemokine (C-C motif) ligand 5 (*Ccl5*), chemokine (C-C motif) ligand 6(*Ccl6*), chemokine (C-C motif) ligand 7 (*Ccl7*), chemokine (C-C motif) ligand 9 (*Ccl9*), chemokine (C-C motif) ligand 12 (*Ccl12*), chemokine (C-C motif) ligand 17 (*Ccl17*), chemokine (C-C motif) ligand 19 (*Ccl19*), chemokine (C-C motif) ligand 20 (*Ccl20*), chemokine (C-C motif) ligand 22 (*Ccl22*),chemokine (C-C motif) ligand 24 (*Ccl24*), chemokine (C-X3-C motif) ligand 1 (*Cx3cl1*), chemokine (C-X-C motif) ligand 1 (melanoma growth stimulating activity, alpha) (*Cxcl1*), chemokine (C-X-C motif) ligand 2 (*Cxcl2*), chemokine (C-X-C motif) ligand 6 (*Cxcl6*), chemokine (C-X-C motif) ligand 9 (*Cxcl9*), chemokine (C-X-C motif) ligand 10 (*Cxcl10*), chemokine (C-X-C motif) ligand 11 (*Cxcl11*), chemokine (C-C motif) receptor 1 (*Ccr1*), chemokine (C-C motif) receptor 2, (*Ccr2*), chemokine (C-C motif) receptor 3(*Ccr3*), chemokine (C-C

motif) receptor 4 (Ccr4), chemokine (C-C motif) receptor 5 (*Ccr5*), chemokine (C-C motif) receptor 6 (*Ccr6*), chemokine (C-C motif) receptor 8 (*Ccr8*), chemokine (C-C motif) receptor 10 (*Ccr10*), Chemokine (C-X3-C motif) receptor 1 (*Cx3cr1*), chemokine (C-X-C motif) receptor 2 (*Cxcr2*), chemokine (C-X-C motif) receptor 3(*Cxcr3*), chemokine (C-X-C motif) receptor 5(*Cxcr5)*

<u>Other</u>: colony stimulating factor 3 (granulocyte) (*Csf3*), oncostatin M (*Osm*), secreted phosphoprotein 1 (*Spp1*),

Table S2. Relative gene expression of chemokines (Mean ± SE). * Significantly different from the control βG- group at the same time point according to the Dunnett post hoc test (* $p < 0.05$, ** $p < 0.01$, *** $p < 0.001$).

**Time**	3 days						7 days					
**Colon inflammation**	*colitis*			control			*colitis*			control		
**Dietary intervention**	ßGl+	ßGh+	ßG-	ßGl+	ßGh+	ßG-	ßGl+	ßGh+	ßG-	ßGl+	ßGh+	ßG-
Ccl2	4,108*	1,705	5,043**	0,666	0,582	0,642	1,711	2,428	1,530	1,484	0,679	1,000
**SE**	1,64	0,61	1,66	0,25	0,12	0,13	0,29	0,83	0,25	0,81	0,05	0,25
Ccl6	2,145	1,461	2,608	1,147	1,105	1,341	2,281	2,394	3,203	1,848	0,989	1,000
**SE**	0,63	0,17	0,91	0,09	0,20	0,14	0,84	0,87	1,41	0,61	0,16	0,11
Ccl7	6,126	2,718	9,581**	0,651	0,479	0,678	2,407	2,587	2,812	2,078	0,813	1,000
**SE**	2,52	1,27	3,35	0,14	0,08	0,12	0,57	0,91	0,36	1,40	0,12	0,10
Ccl9	0,214	0,153	0,193	0,199	0,320	0,130	0,535	0,514	0,535	0,668	0,265	1,000
**SE**	0,08	0,04	0,05	0,05	0,16	0,02	0,24	0,18	0,22	0,24	0,20	0,38
Ccl11	1,296	1,519	3,381	1,552	0,757	1,241	2,108	1,539	1,305	1,251	1,711	1,000
**SE**	0,27	0,16	1,75	0,28	0,19	0,24	0,38	0,23	0,23	0,26	0,49	0,26
Ccl12	21,583	10,616	17,314	0,792	1,039	1,046	14,206	16,178	25,334*	1,540	2,065	1,000
**SE**	5,52	3,75	7,11	0,20	0,22	0,24	5,14	6,41	11,99	0,73	1,03	0,25
Ccl17	1,475***	2,767***	1,537	0,378	0,821	0,698	5,482	5,396	3,534	1,605	0,454	1,000
**SE**	0,17	0,64	0,32	0,07	0,29	0,25	1,29	1,65	0,36	0,74	0,02	0,31
Ccl19	0,023	0,016	0,220	0,041	0,010	0,015	0,499	0,273	0,416	0,635	0,485	1,000
**SE**	0,01	0,00	0,21	0,03	0,00	0,00	0,28	0,26	0,40	0,39	0,48	0,41
Ccl20	0,310	0,386	0,207	0,257	0,616	0,355	0,327	0,752	0,263	0,765	0,247	1,000
**SE**	0,13	0,09	0,08	0,03	0,17	0,12	0,18	0,42	0,07	0,28	0,05	0,48
Ccl22	0,663	1,041	0,229	0,809	3,007	1,098	1,887	2,515	1,880	1,566	0,571	1,000
**SE**	0,37	0,23	0,06	0,14	1,46	0,35	0,80	1,11	0,67	0,60	0,20	0,35
Ccl24	0,901	1,105	0,569	1,462	0,923	0,888	0,769	0,884	0,739	1,108	0,852	1,000
**SE**	0,23	0,06	0,16	0,29	0,09	0,12	0,15	0,22	0,20	0,09	0,21	0,13
Cxcl6	15,405	3,969	23,984*	0,591	0,389	0,665	2,599	2,504	2,598	1,773	0,477	1,000
**SE**	9,34	1,83	12,04	0,18	0,09	0,29	0,44	0,37	1,03	0,72	0,08	0,24
Cxcl12	0,974	1,061	1,406	1,538	1,218	1,082	0,741	0,977	1,084	1,131	1,199	1,000
**SE**	0,29	0,16	0,12	0,23	0,19	0,16	0,20	0,33	0,30	0,08	0,28	0,21
Cx3cl1	0,982	0,200	0,084	1,722	1,630	0,808	0,091	0,438	0,207	1,173	0,963	1,000
**SE**	0,63	0,02	0,02	0,83	0,68	0,61	0,03	0,28	0,14	0,59	0,82	0,46

Table S3. Relative gene expression of chemokine receptors (Mean ± SE). * Significantly different from the control βG- group at the same time point according to the Dunnett post hoc test (** $p < 0.01$, *** $p < 0.001$).

**Time**	3 days						7 days					
**Colon inflammation**	*colitis*			control			*colitis*			control		
**Dietary intervention**	ßGl+	ßGh+	ßG-	ßGl+	ßGh+	ßG-	ßGl+	ßGh+	ßG-	ßGl+	ßGh+	ßG-
Ccr3	1,559	2,184	2,536	1,295	0,993	1,242	3,143***	3,026**	3,122***	1,167	0,917	1,000
**SE**	0,38	0,52	0,35	0,14	0,20	0,14	0,49	0,58	0,19	0,23	0,20	0,18
Ccr4	0,827	0,593	1,300	0,377	0,312	0,264	1,129	1,338	0,869	1,147	0,634	1,000
**SE**	0,34	0,20	0,94	0,10	0,06	0,04	0,43	0,30	0,44	0,38	0,59	0,46
Ccr6	0,088	0,088	0,132	0,126	0,103	0,098	0,682	0,800	0,396	0,654	0,294	1,000
**SE**	0,02	0,02	0,06	0,04	0,06	0,01	0,34	0,40	0,15	0,30	0,19	0,42
Ccr8	1,656	1,139	1,627	0,786	1,823	0,435	1,336	2,443	1,216	0,624	0,783	1,000
**SE**	0,70	0,32	0,78	0,16	1,24	0,31	0,40	1,20	0,59	0,39	0,00	0,48
Ccr10	0,763	0,757	0,728	2,122	1,618	1,246	0,787	0,755	0,743	1,212	0,813	1,000
**SE**	0,37	0,12	0,29	0,46	0,24	0,24	0,18	0,18	0,24	0,44	0,38	0,26
Cxcr1	1,533	0,978	2,026	1,853	1,076	1,485	1,845	1,772	0,935	1,415	1,010	1,000
**SE**	0,38	0,29	0,58	0,37	0,18	0,34	0,48	0,33	0,19	0,41	0,22	0,47
Cxcr3	0,622	0,599	0,990	0,613	0,788	0,300	0,925	0,857	0,743	0,795	0,774	1,000
**SE**	0,39	0,21	0,61	0,15	0,33	0,12	0,27	0,15	0,23	0,19	0,57	0,35
Cxcr5	0,048	0,115	0,222	0,115	0,096	0,057	0,123	0,071	0,132	0,557	0,019	1,000
**SE**	0,01	0,08	0,16	0,03	0,03	0,02	0,05	0,03	0,07	0,32	0,00	0,45
Cx3cr1	0,989	0,826	1,052	0,719	0,557	0,713	0,995	0,967	0,659	0,616	0,523	1,000
**SE**	0,36	0,04	0,20	0,10	0,16	0,10	0,20	0,20	0,10	0,05	0,20	0,25
